# Supplementary material for: Self-Referenced Multifrequency Phase-Resolved Luminescence Spectroscopy
Source: Sensors (Basel). 2020 Sep 24;20(19):5482. doi: 10.3390/s20195482 (PMC7583794; doi:10.3390/s20195482)
Supplement: Supplementary file 1 [file sensors-20-05482-s001.pdf]

# Supplementary Materials: Supplementary Materials: Self-Referenced Multifrequency Phase-Resolved Luminescence Spectroscopy

**Angel de la Torre [1], Santiago Medina-Rodríguez [2], Jose C. Segura [1], and Jorge F. Fernández-Sánchez [3,*]**

## Contents


**1 Function $F_n(x)$ 2**

**2 Simulation of a monoexponential luminescent system with $M_0 = 1$, $\tau_0 = 100\mu$s and $k = 0.5$ kPa$^{-1}$ 3**

**3 Experimental data: modulation factor and phase-shift at each harmonic 7**

**4 One-site and two-sites models fitting the experimental data 8**
4.1 One-site model fitting the experimental data . . . . . 8
4.2 Two-sites model fitting the experimental data . . . . . 9

**5 Simulation of the one-site model fitting the experimental data 10**

**6 Simulation of the two-sites model fitting the experimental data 13**

**7 Experimental lifetime estimations (conventional procedure) 18**
7.1 Modulation factor and phase-shift measurements . . . . . 18
7.2 Modulation factor and phase-shift based lifetime estimations . . . . . 21

**8 Self-referenced amplitudes 24**

**9 Self-referenced delays 28**

**10 Self-referenced apparent lifetimes 31**

**11 Calibration parameters for each oxygen determination 34**
11.1 Parameters of the Demas models providing the curves $C(\tau)$ . . . . . 34
11.2 Uncertainties for each oxygen determination . . . . . 35

**12 RMS error in oxygen determination (evaluation partition) 37**
12.1 Not combined oxygen determination . . . . . 37
12.2 Combined oxygen determination . . . . . 40

## 1. Function $F_n(x)$

The function $F_n(x)$ providing the relationship between the delay of the $n$-th harmonic and the lifetime (equation (20) in the manuscript):

$$\Delta t_{em}(n) - \Delta t_{exc}(n) = \frac{1}{n\,\omega_0}(2\pi p + F_n(\omega_0 \tau_q)) \tag{1}$$

is defined as:

$$F_n(x) \equiv \arctan(n\,x) - n\arctan(x) \tag{2}$$

and is represented in the following plots for different values of the harmonic index $n$.

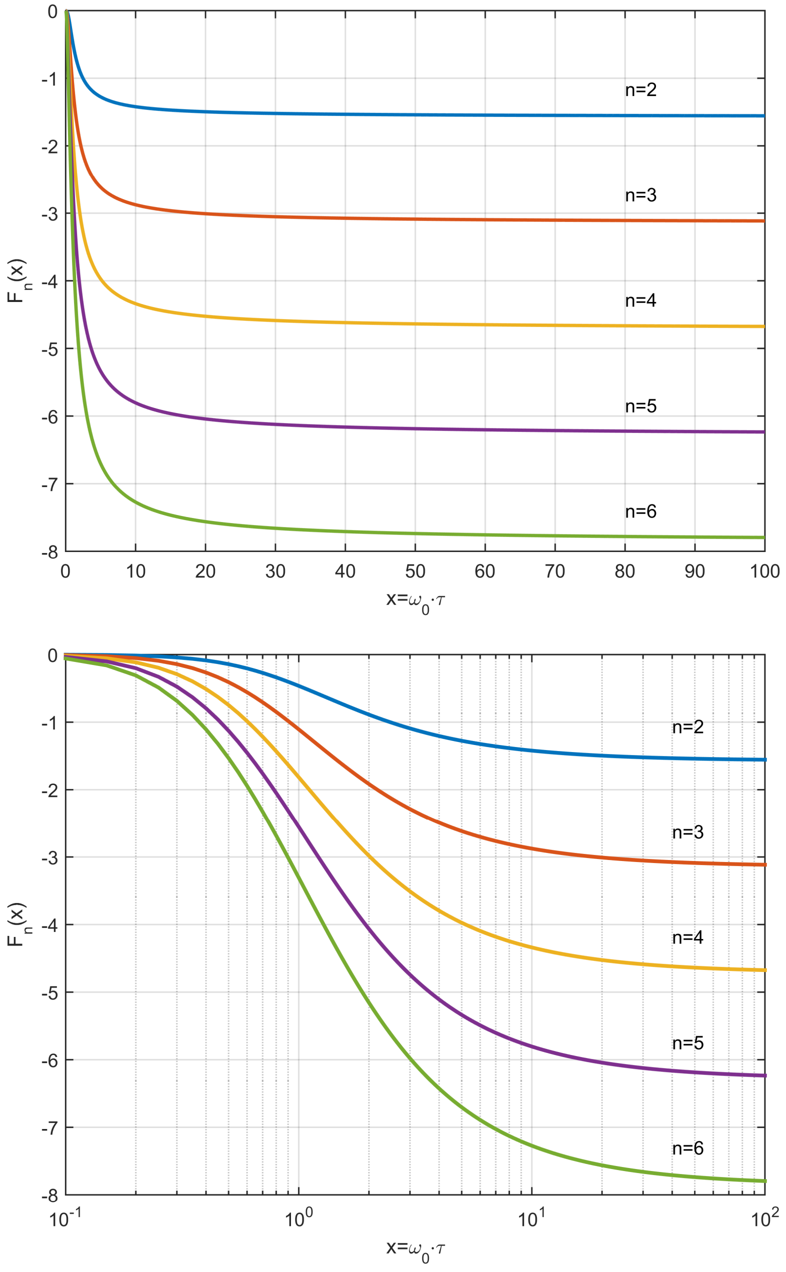


**Figure S1.** Funcion $F_n(x)$ for $n = 2, \ldots, 6$, represented with $x$ in linear (top) and logarithmic (bottom) scales.

## 2. Simulation of a monoexponential luminescent system with $M_0 = 1$, $\tau_0 = 100\mu$s and $k = 0.5$ kPa$^{-1}$

This section includes figures corresponding to the simulation of a monoexponential luminescent system with parameters $M_0 = 1$, $\tau_0 = 100\mu$s and $k = 0.5$ kPa$^{-1}$ excited with a periodic repetition of rectangular pulses of amplitude 1.0 (arbitrary units), fundamental frequency 1 kHz, and 10% duty cycle. The simulation has been performed at concentrations $C = 0$ and $C = 5$ kPa (with lifetimes 100 and 28.5 $\mu$s, respectively).

The figures represent the excitation and emission signals, the corresponding harmonic decomposition, the estimation of the self-referenced normalized amplitudes and delays (referenced to the first harmonic) and the corresponding estimated lifetimes.

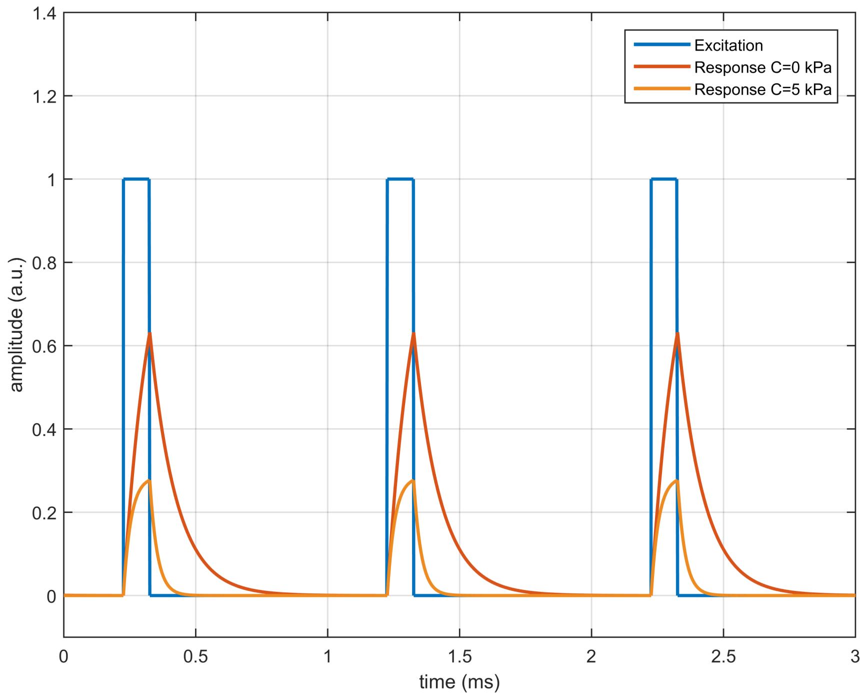


**Figure S2.** Simulated response of a first order luminescent system with $M_0 = 1$, $\tau_0 = 100\mu$s and $k = 0.5$ kPa$^{-1}$ excited with rectangular pulses of amplitude 1.0 a.u. presented at 1 kHz with 10% duty cycle, for quencher concentrations $C = 0$ and $C = 5$ kPa.

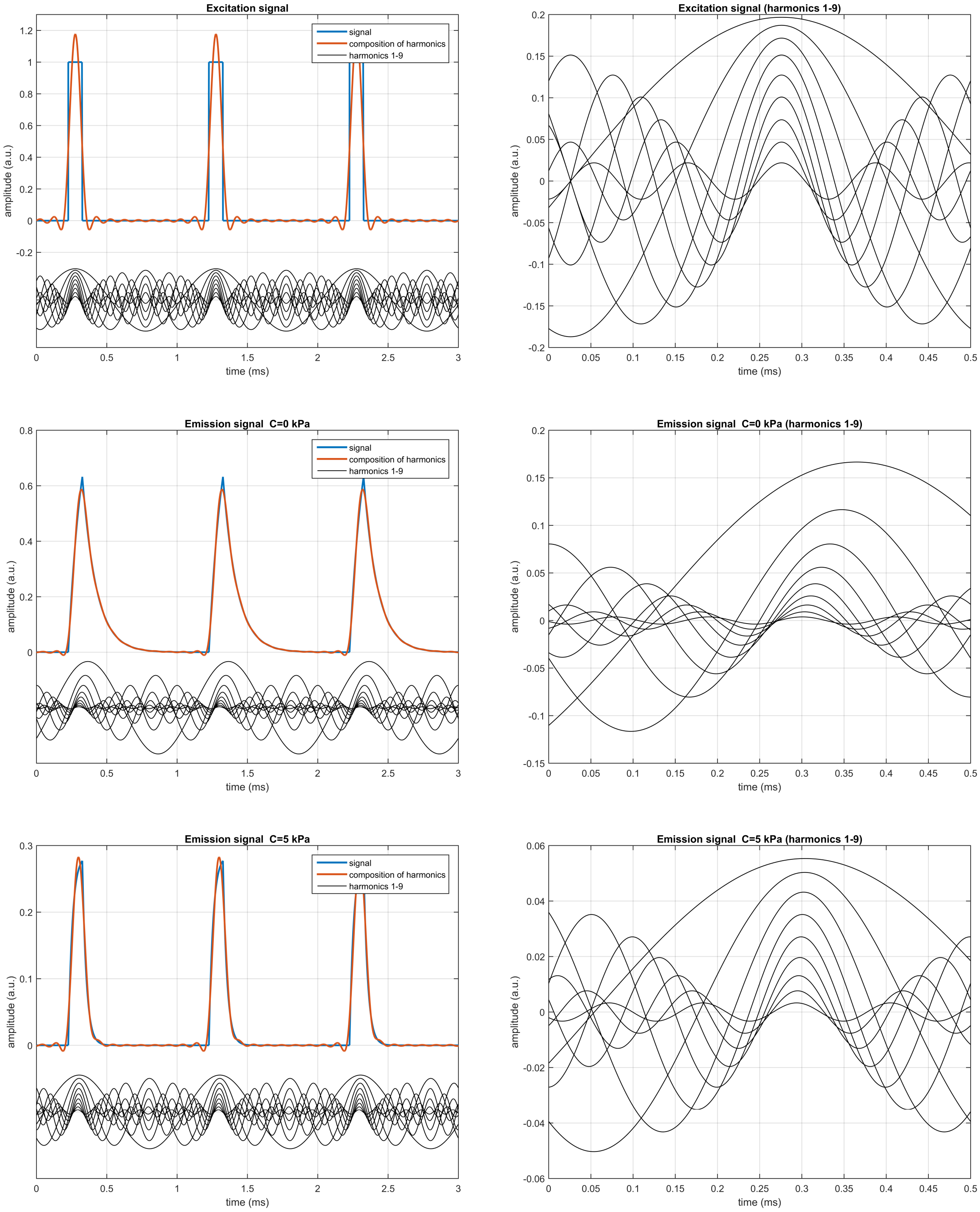


**Figure S3.** Excitation signal (top) and emission signals at $C = 0$ kPa (center) and at $C = 5$ kPa (bottom). The left panels represent the excitation or emission signals, the harmonics (from 1st to 9th) and the composition of the harmonics. The right panels show a detail of the harmonics for each signal. Simulation of monoexponential system ($M_0 = 1$, $\tau_0 = 100\mu$s and $k = 0.5$ kPa$^{-1}$).

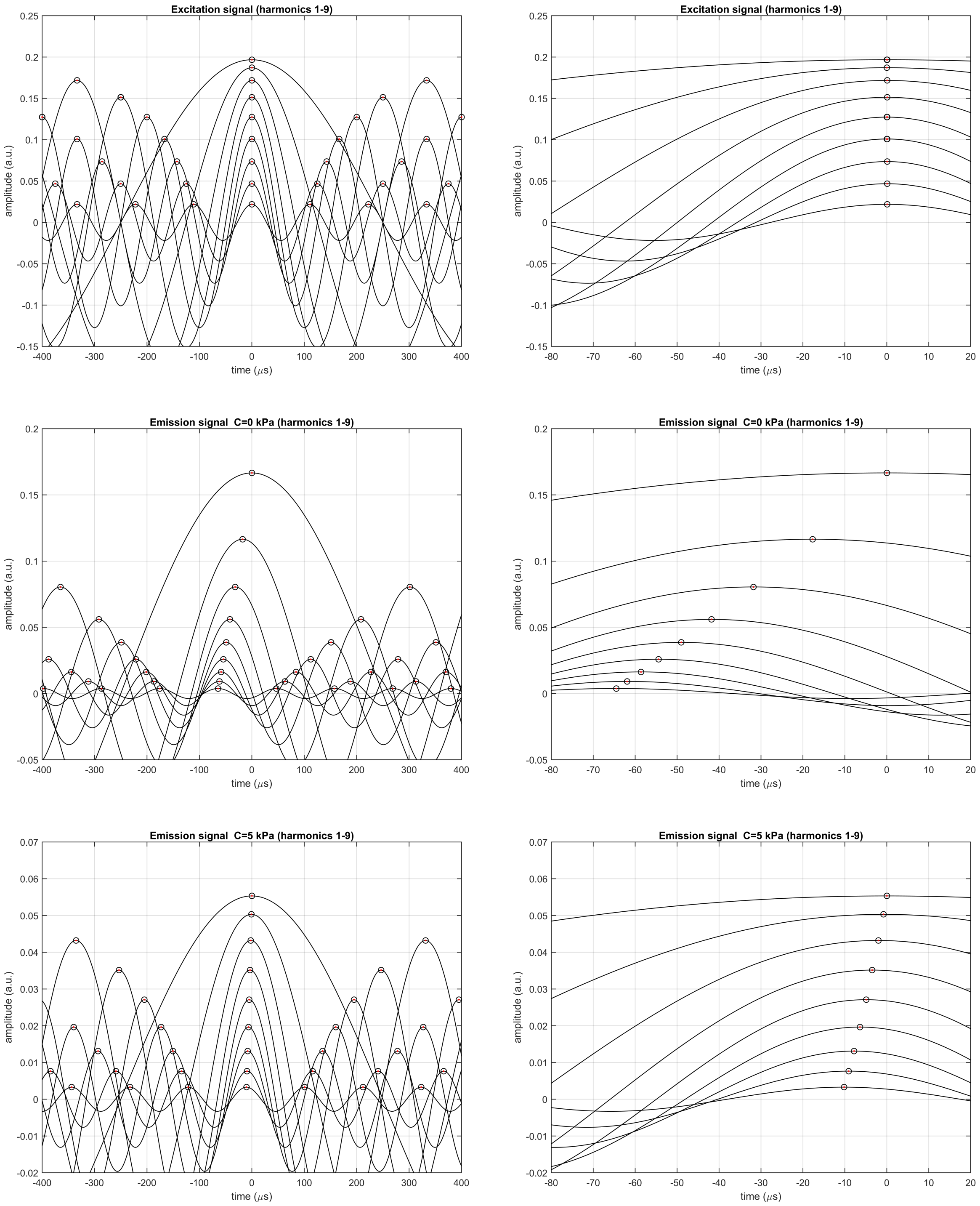


**Figure S4.** Harmonics of the excitation signal (top) and emission signals at $C = 0$ kPa (center) and at $C = 5$ kPa (bottom). Identification of the local maxima of the different harmonics. Simulation of monoexponential system ($M_0 = 1$, $\tau_0 = 100\mu$s and $k = 0.5$ kPa$^{-1}$).

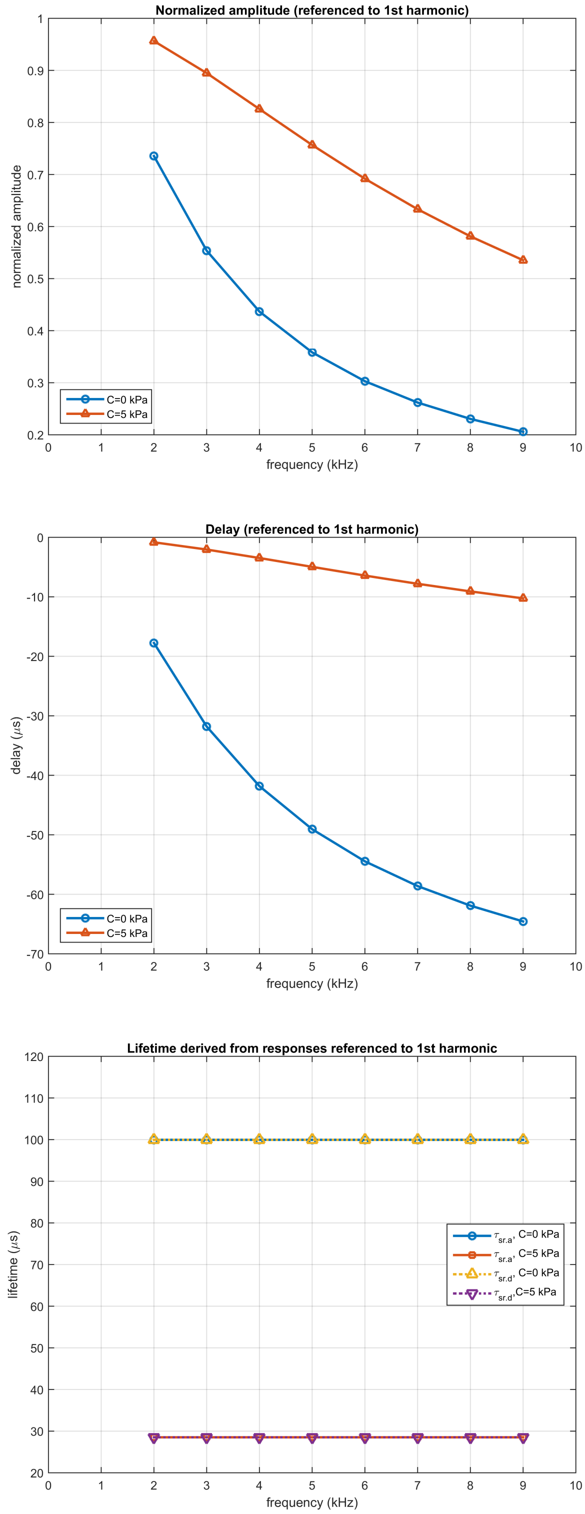


**Figure S5.** Self-referenced normalized amplitude (top), delay (center) and corresponding estimated lifetimes (bottom). Simulation of monoexponential system ($M_0 = 1$, $\tau_0 = 100\mu$s and $k = 0.5$ kPa$^{-1}$).

## 3. Experimental data: modulation factor and phase-shift at each harmonic

| **Modulation factor $m(n)$ (arbitrary units)** | | | | | | | |
|---|---|---|---|---|---|---|---|
| $C$ (kPa) | $f$=1715 Hz ($n$=1) | $f$=3430 Hz ($n$=2) | $f$=5145 Hz ($n$=3) | $f$=6860 Hz ($n$=4) | $f$=8575 Hz ($n$=5) | $f$=10290 Hz ($n$=6) | $f$=12005 Hz ($n$=7) |
| 0.00 | 1.8226 (0.0030) | 1.2841 (0.0028) | 0.9592 (0.0030) | 0.7499 (0.0036) | 0.6241 (0.0037) | 0.5321 (0.0046) | 0.4622 (0.0063) |
| 0.50 | 1.6586 (0.0029) | 1.2180 (0.0025) | 0.9306 (0.0026) | 0.7365 (0.0033) | 0.6177 (0.0033) | 0.5289 (0.0047) | 0.4609 (0.0060) |
| 1.00 | 1.5211 (0.0023) | 1.1544 (0.0022) | 0.9015 (0.0023) | 0.7231 (0.0030) | 0.6098 (0.0032) | 0.5239 (0.0038) | 0.4572 (0.0050) |
| 2.00 | 1.2955 (0.0026) | 1.0303 (0.0024) | 0.8340 (0.0026) | 0.6868 (0.0034) | 0.5878 (0.0030) | 0.5094 (0.0036) | 0.4477 (0.0046) |
| 3.00 | 1.1286 (0.0023) | 0.9254 (0.0022) | 0.7699 (0.0022) | 0.6500 (0.0032) | 0.5635 (0.0028) | 0.4928 (0.0032) | 0.4356 (0.0045) |
| 4.00 | 1.0006 (0.0027) | 0.8382 (0.0023) | 0.7126 (0.0025) | 0.6139 (0.0032) | 0.5391 (0.0026) | 0.4757 (0.0031) | 0.4230 (0.0047) |
| 5.00 | 0.8997 (0.0022) | 0.7653 (0.0020) | 0.6616 (0.0020) | 0.5795 (0.0030) | 0.5151 (0.0026) | 0.4581 (0.0032) | 0.4099 (0.0045) |
| 6.00 | 0.8183 (0.0022) | 0.7042 (0.0020) | 0.6172 (0.0020) | 0.5491 (0.0028) | 0.4926 (0.0024) | 0.4412 (0.0030) | 0.3972 (0.0041) |
| 7.00 | 0.7514 (0.0020) | 0.6525 (0.0019) | 0.5784 (0.0019) | 0.5212 (0.0027) | 0.4718 (0.0022) | 0.4249 (0.0026) | 0.3846 (0.0037) |
| 8.00 | 0.6955 (0.0021) | 0.6085 (0.0019) | 0.5444 (0.0020) | 0.4963 (0.0024) | 0.4523 (0.0024) | 0.4101 (0.0030) | 0.3729 (0.0041) |
| 9.00 | 0.6478 (0.0020) | 0.5699 (0.0020) | 0.5141 (0.0019) | 0.4736 (0.0024) | 0.4344 (0.0024) | 0.3956 (0.0028) | 0.3613 (0.0038) |
| 10.00 | 0.6067 (0.0019) | 0.5364 (0.0017) | 0.4873 (0.0020) | 0.4529 (0.0021) | 0.4176 (0.0022) | 0.3815 (0.0027) | 0.3491 (0.0044) |
| 12.00 | 0.5380 (0.0018) | 0.4792 (0.0017) | 0.4408 (0.0018) | 0.4167 (0.0021) | 0.3883 (0.0020) | 0.3575 (0.0024) | 0.3301 (0.0034) |

**Table S1.** Modulation factor (in arbitrary units) for each harmonic at different concentrations. Mean and standard deviation (in parentheses) for the calibration partition (estimated with 125 measurements at each concentration).

| **Phase shift $\phi(n)$ (degrees)** | | | | | | | |
|---|---|---|---|---|---|---|---|
| $C$ (kPa) | $f$=1715 Hz ($n$=1) | $f$=3430 Hz ($n$=2) | $f$=5145 Hz ($n$=3) | $f$=6860 Hz ($n$=4) | $f$=8575 Hz ($n$=5) | $f$=10290 Hz ($n$=6) | $f$=12005 Hz ($n$=7) |
| 0.00 | -35.954 (0.060) | -54.045 (0.111) | -63.513 (0.163) | -67.883 (0.243) | -72.008 (0.334) | -75.252 (0.477) | -77.768 (0.791) |
| 0.50 | -32.583 (0.051) | -50.372 (0.096) | -60.231 (0.154) | -65.134 (0.255) | -69.656 (0.302) | -73.263 (0.423) | -76.062 (0.723) |
| 1.00 | -29.928 (0.053) | -47.135 (0.093) | -57.153 (0.159) | -62.494 (0.275) | -67.451 (0.346) | -71.317 (0.526) | -74.379 (0.777) |
| 2.00 | -26.053 (0.055) | -41.924 (0.103) | -51.779 (0.171) | -57.576 (0.309) | -63.126 (0.344) | -67.438 (0.492) | -70.809 (0.765) |
| 3.00 | -23.353 (0.058) | -37.968 (0.105) | -47.328 (0.160) | -53.302 (0.293) | -59.241 (0.287) | -63.909 (0.381) | -67.519 (0.638) |
| 4.00 | -21.340 (0.060) | -34.861 (0.091) | -43.652 (0.143) | -49.675 (0.256) | -55.880 (0.263) | -60.778 (0.402) | -64.651 (0.604) |
| 5.00 | -19.787 (0.075) | -32.375 (0.110) | -40.574 (0.155) | -46.480 (0.263) | -52.855 (0.250) | -57.947 (0.376) | -61.958 (0.581) |
| 6.00 | -18.544 (0.073) | -30.298 (0.118) | -37.918 (0.180) | -43.647 (0.301) | -50.176 (0.290) | -55.340 (0.398) | -59.447 (0.602) |
| 7.00 | -17.528 (0.083) | -28.581 (0.120) | -35.654 (0.162) | -41.308 (0.279) | -47.875 (0.297) | -53.139 (0.438) | -57.339 (0.654) |
| 8.00 | -16.663 (0.071) | -27.097 (0.112) | -33.680 (0.183) | -39.241 (0.315) | -45.790 (0.314) | -51.079 (0.450) | -55.310 (0.647) |
| 9.00 | -15.929 (0.082) | -25.821 (0.118) | -31.936 (0.178) | -37.351 (0.310) | -43.987 (0.304) | -49.287 (0.419) | -53.592 (0.576) |
| 10.00 | -15.294 (0.080) | -24.714 (0.107) | -30.369 (0.175) | -35.668 (0.241) | -42.306 (0.304) | -47.607 (0.417) | -51.847 (0.622) |
| 12.00 | -14.218 (0.085) | -22.764 (0.116) | -27.584 (0.178) | -32.700 (0.296) | -39.357 (0.284) | -44.656 (0.406) | -49.032 (0.638) |

**Table S2.** Phase shift (in degrees) for each harmonic at different concentrations. Mean and standard deviation (in parentheses) for the calibration partition (estimated with 125 measurements at each concentration).

## 4. One-site and two-sites models fitting the experimental data

### 4.1. One-site model fitting the experimental data

The one-site model describes the luminescence as a function of the quencher concentration according to the following frequency response:

$$H(j\omega) = \frac{M_0}{1 + kC + j\omega\tau_0} \quad (3)$$

where the modulus and the argument of this complex frequency response correspond, respectively, to the modulation factor and the phase shift. The experimental data in the calibration partition have been used to fit the parameters of the one-site model.

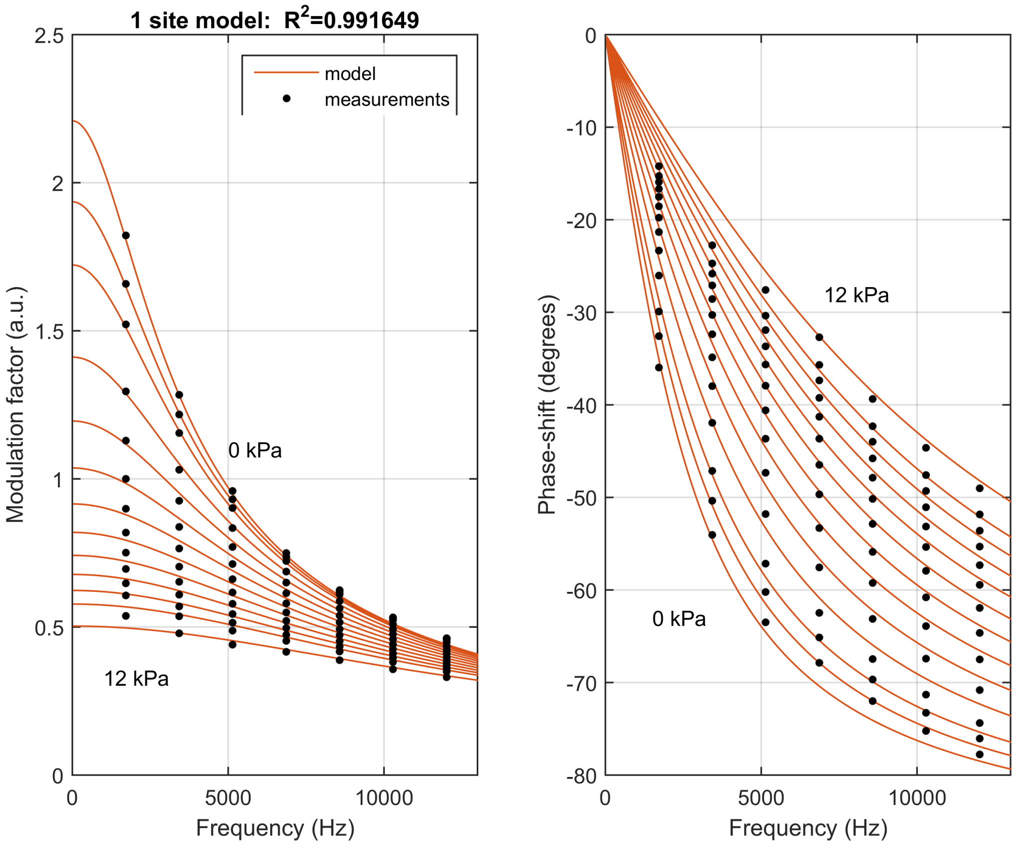


| model parameters: | |
|---|---|
| $M_0$ | 2.2089 a.u. |
| $\tau_0$ | 65.0889 $\mu$s |
| $k$ | 0.2826 kPa$^{-1}$ |
| $R^2$ | 0.991649 |

**Figure S6.** Fitting of the experimental data with a one-site model.

## *4.2. Two-sites model fitting the experimental data*

The two-sites model describes the luminescence as a function of the quencher concentration according to the following frequency response:

$$H(j\omega) = \frac{M_{0,1}}{1 + k_1\, C + j\omega\tau_{0,1}} + \frac{M_{0,2}}{1 + k_2\, C + j\omega\tau_{0,2}} \tag{4}$$

where the modulus and the argument of this complex frequency response correspond, respectively, to the modulation factor and the phase shift. The experimental data in the calibration partition have been used to fit the parameters of the two-sites model.

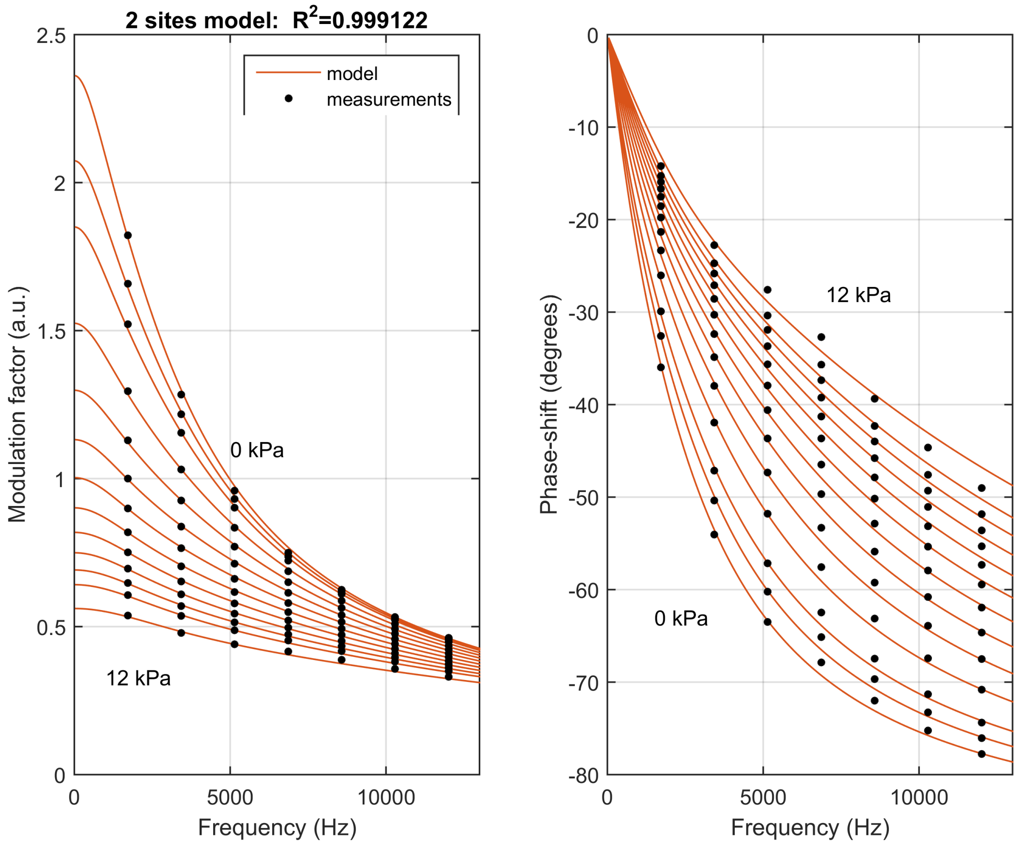


| model parameters: | |
|---|---|
| $M_{0,1}$ | 1.7709 a.u. |
| $\tau_{0,1}$ | 56.4362 $\mu$s |
| $k_1$ | 0.3138 kPa$^{-1}$ |
| $M_{0,2}$ | 0.5908 a.u. |
| $\tau_{0,2}$ | 139.8189 $\mu$s |
| $k_2$ | 0.1763 kPa$^{-1}$ |
| $R^2$ | 0.999122 |

**Figure S7.** Fitting of the experimental data with a two-sites model.

## 5. Simulation of the one-site model fitting the experimental data

This section includes figures corresponding to the simulation of a monoexponential luminescent system with parameters $M_0 = 2.2089$ a.u., $\tau_0 = 65.0889\mu$s and $k = 0.2826$ kPa$^{-1}$ (i.e. those obtained for the one-site model fitting the experimental data) excited with a periodic repetition of rectangular pulses of amplitude 1.0 (arbitrary units), fundamental frequency 1715 Hz, and 10% duty cycle (i.e. the excitation configuration used in the experiments). The simulation has been performed at concentrations $C = 0.5$ and $C = 10$ kPa (with lifetimes 56.98 and 16.96 $\mu$s, respectively).

The figures represent the excitation and emission signals, the corresponding harmonic decomposition, the estimation of the self-referenced normalized amplitudes and delays (referenced to the first harmonic) and the corresponding estimated lifetimes.

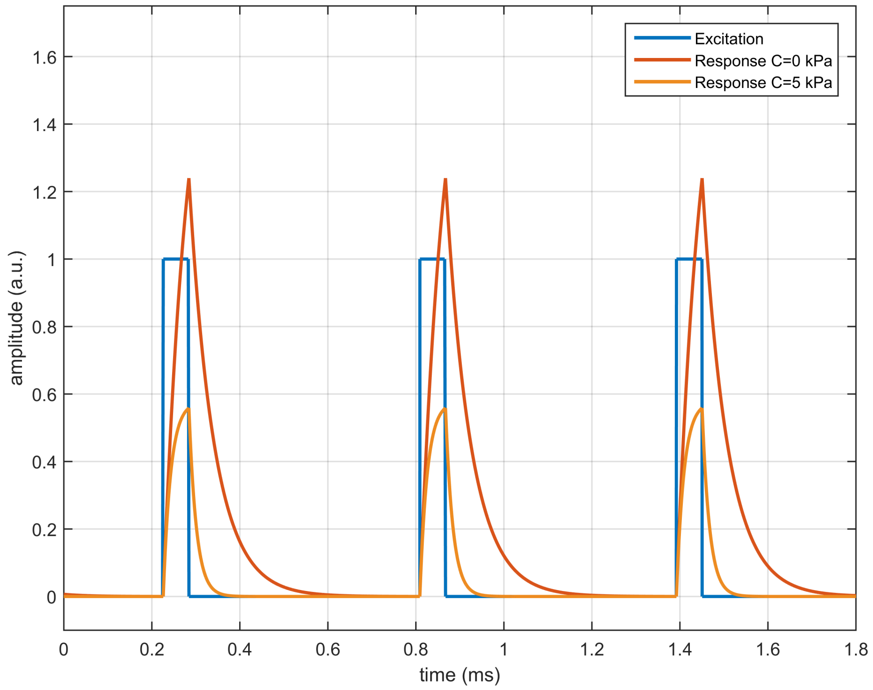


**Figure S8.** Simulated response of the one-site model fitting the experimental data (first order luminescent system with $M_0 = 2.2089$, $\tau_0 = 65.0889\mu$s and $k = 0.2826$ kPa$^{-1}$) excited with rectangular pulses of amplitude 1.0 a.u. presented at 1715 Hz with 10% duty cycle, for quencher concentrations $C = 0.5$ and $C = 10$ kPa.

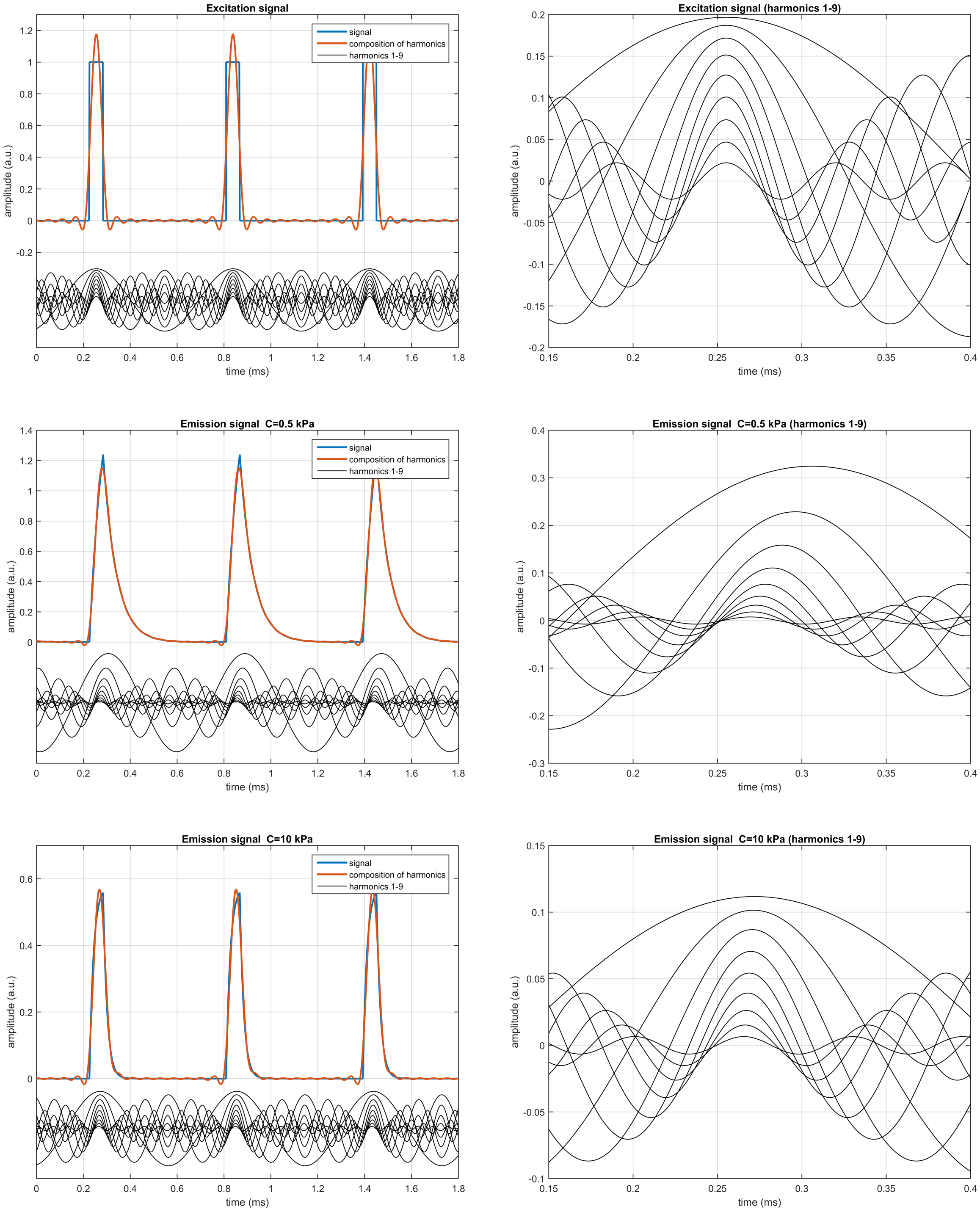


**Figure S9.** Excitation signal (top) and emission signals at $C = 0.5$ kPa (center) and at $C = 10$ kPa (bottom). The left panels represent the excitation or emission signals, the harmonics (from 1st to 9th) and the composition of the harmonics. The right panels show a detail of the harmonics for each signal. Simulation of the one-site model fitting the experimental data.

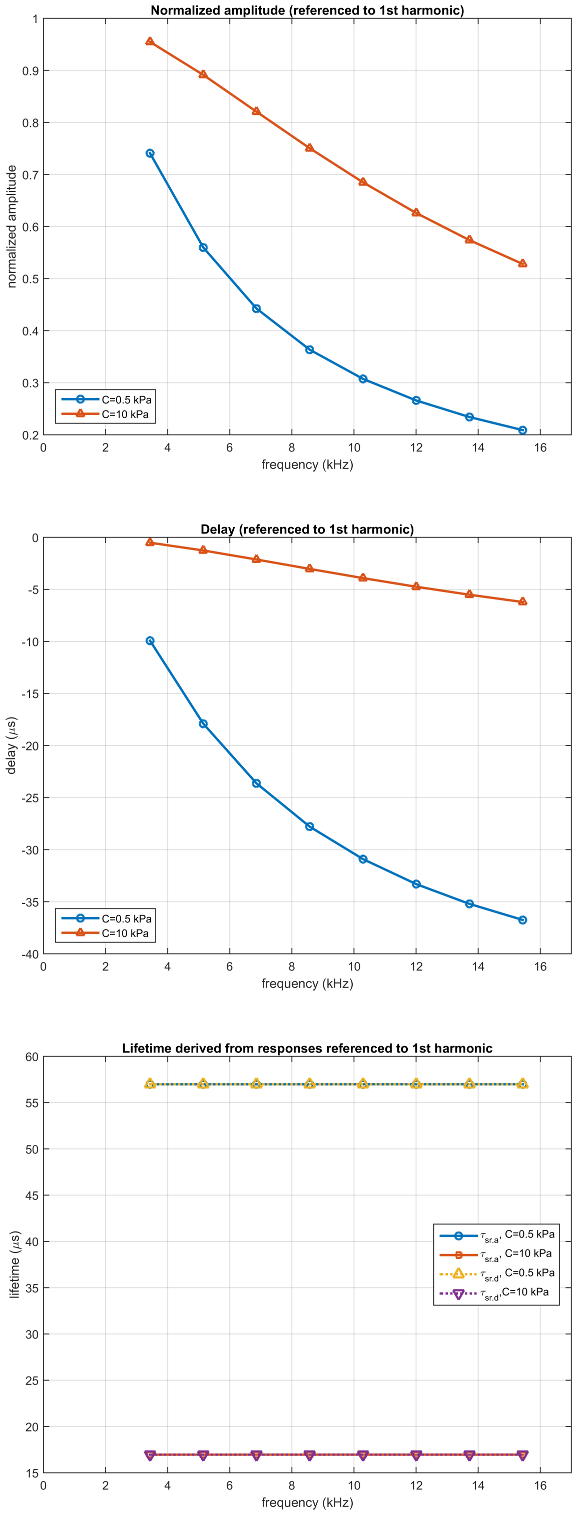


**Figure S10.** Self-referenced normalized amplitude (top), delay (center) and corresponding estimated lifetimes (bottom). Simulation of the one-site model fitting the experimental data.

## 6. Simulation of the two-sites model fitting the experimental data

This section includes figures corresponding to the simulation of a two-sites luminescent system with parameters $M_{0,1} = 1.7709$ a.u., $\tau_{0,1} = 56.4362\mu$s and $k_1 = 0.3138$ kPa$^{-1}$, $M_{0,2} = 0.5908$ a.u., $\tau_{0,2} = 139.8189\mu$s and $k_2 = 0.1763$ kPa$^{-1}$ (i.e. those obtained for the two-sites model fitting the experimental data) excited with a periodic repetition of rectangular pulses of amplitude 1.0 (arbitrary units), fundamental frequency 1715 Hz, and 10% duty cycle (i.e. the excitation configuration used in the experiments). The simulation has been performed at concentrations $C = 0.5$ and $C = 10$ kPa (no lifetimes defined for a non-monoexponential system; apparent lifetimes expected to depend on the estimation procedure).

The figures represent the excitation and emission signals, the corresponding harmonic decomposition, the estimation of the self-referenced normalized amplitudes and delays (referenced to the first harmonic) and the corresponding estimated apparent lifetimes.

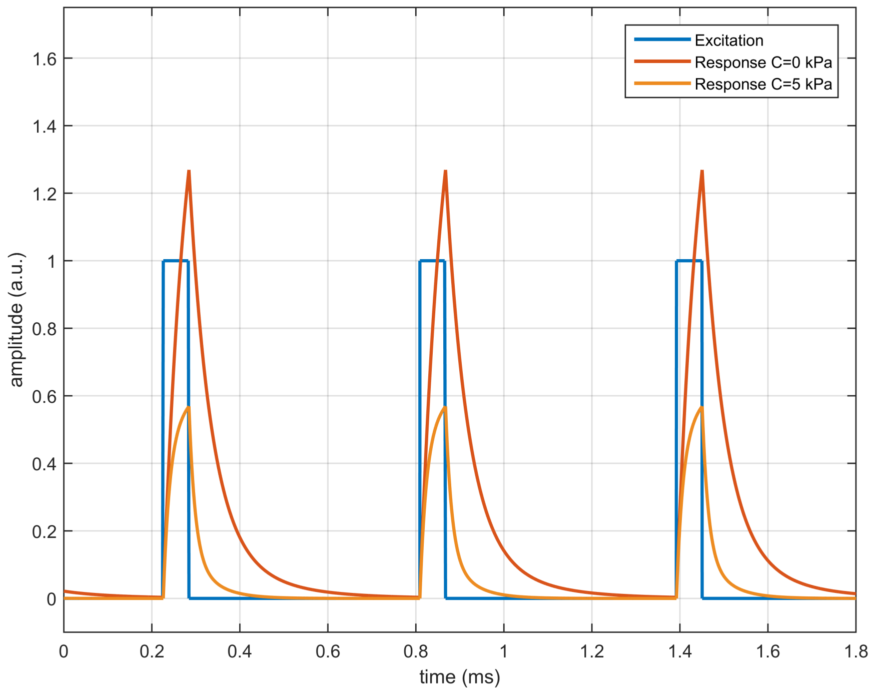


**Figure S11.** Simulated response of the two-sites model fitting the experimental data (two-sites luminescent system with $M_{0,1} = 1.7709$ a.u., $\tau_{0,1} = 56.4362\mu$s and $k_1 = 0.3138$ kPa$^{-1}$, $M_{0,2} = 0.5908$ a.u., $\tau_{0,2} = 139.8189\mu$s and $k_2 = 0.1763$ kPa$^{-1}$) excited with rectangular pulses of amplitude 1.0 a.u. presented at 1715 Hz with 10% duty cycle, for quencher concentrations $C = 0.5$ and $C = 10$ kPa.

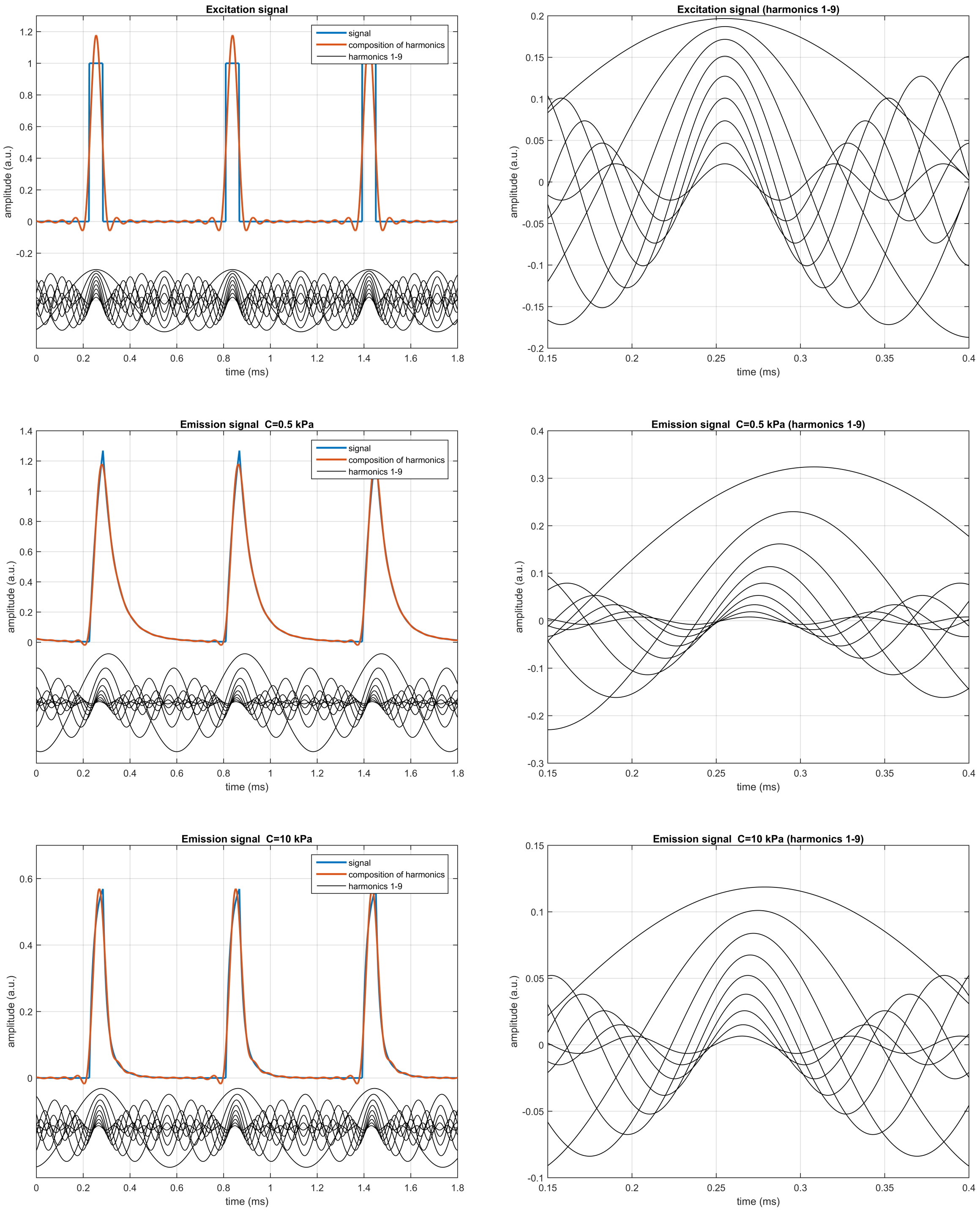


**Figure S12.** Excitation signal (top) and emission signals at $C = 0.5$ kPa (center) and at $C = 10$ kPa (bottom). The left panels represent the excitation or emission signals, the harmonics (from 1st to 9th) and the composition of the harmonics. The right panels show a detail of the harmonics for each signal. Simulation of the two-sites model fitting the experimental data.

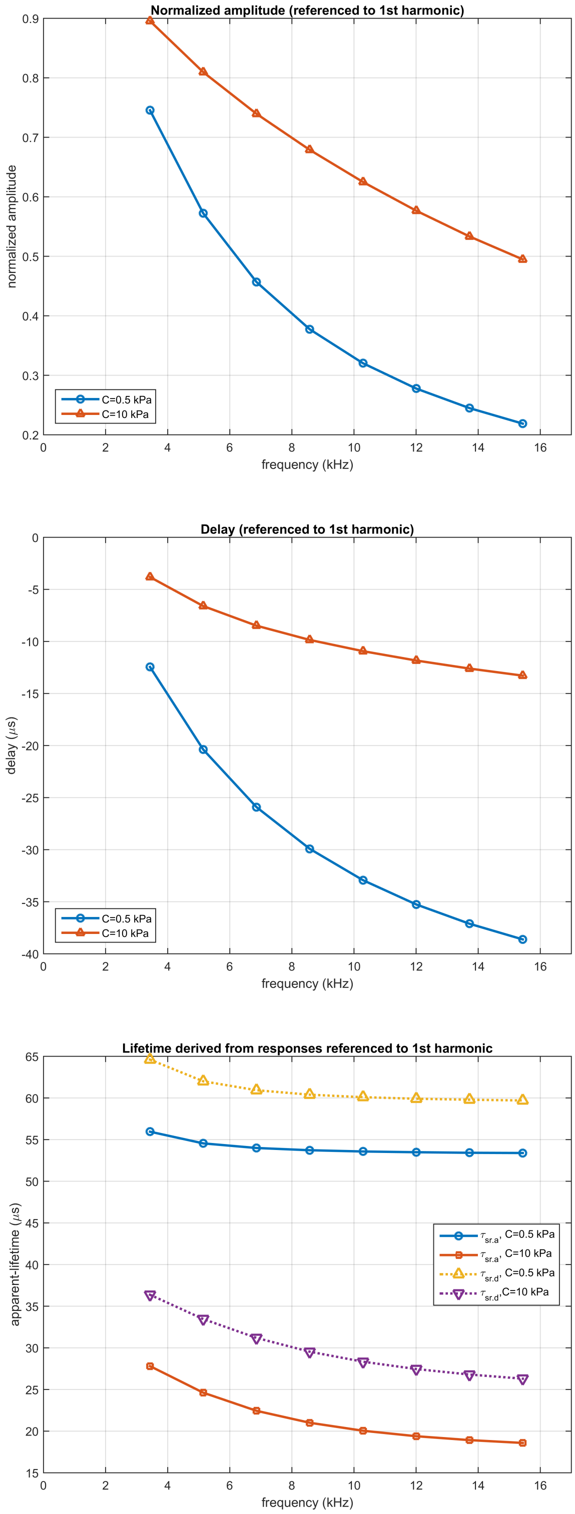


**Figure S13.** Self-referenced normalized amplitude (top), delay (center), and corresponding estimated apparent lifetimes (bottom). Simulation of the two-sites model fitting the experimental data.

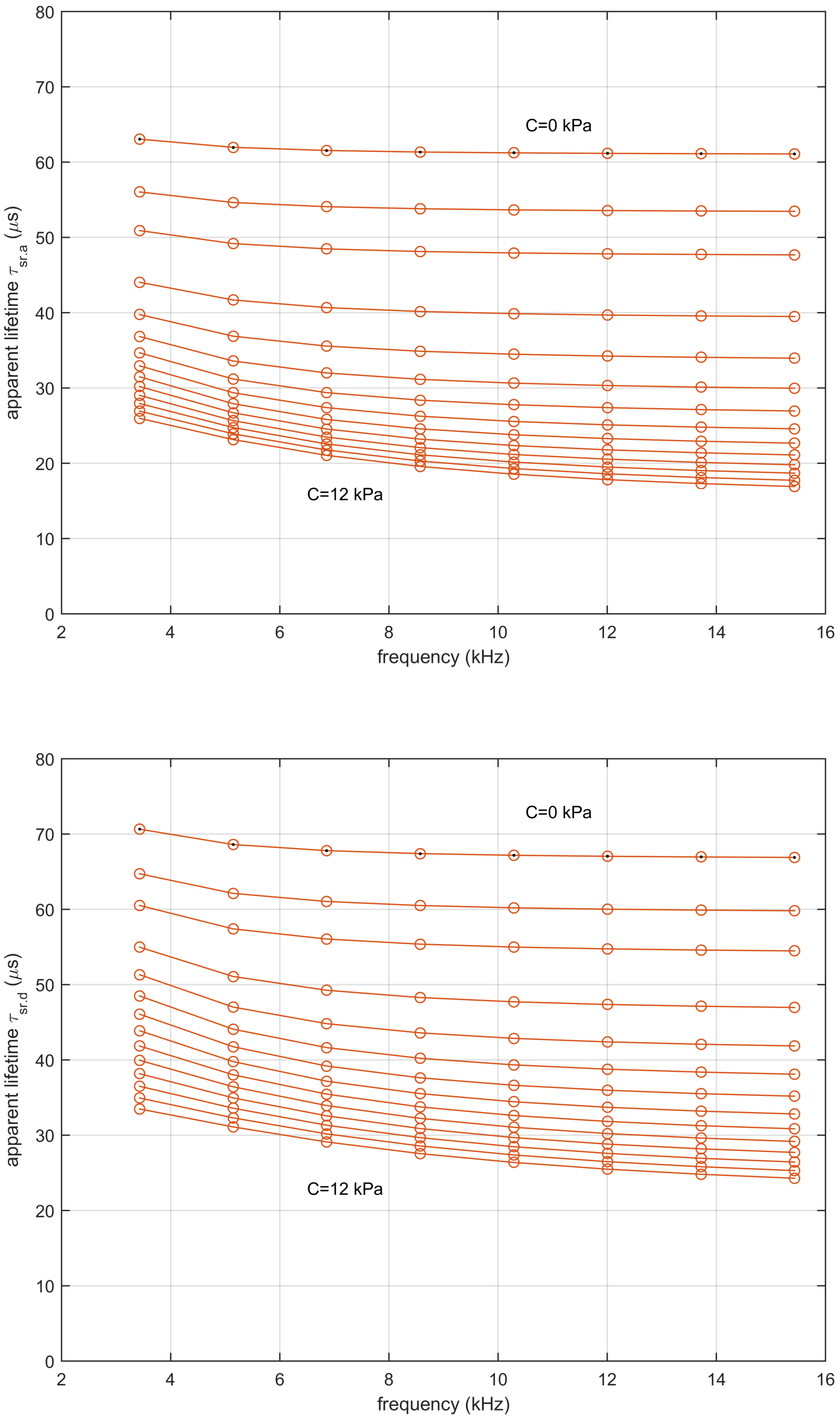


**Figure S14.** Estimated apparent lifetimes as a function of the harmonic frequency for the simulated two-sites model fitting the experimental data. Apparent lifetimes estimated at the following concentrations: 0, 0.5, 1, 2, 3, 4, 5, 6, 7, 8, 9, 10, 11 and 12 kPa. Apparent lifetimes based on self-referenced normalized amplitudes (top panel) and delays (bottom panel).

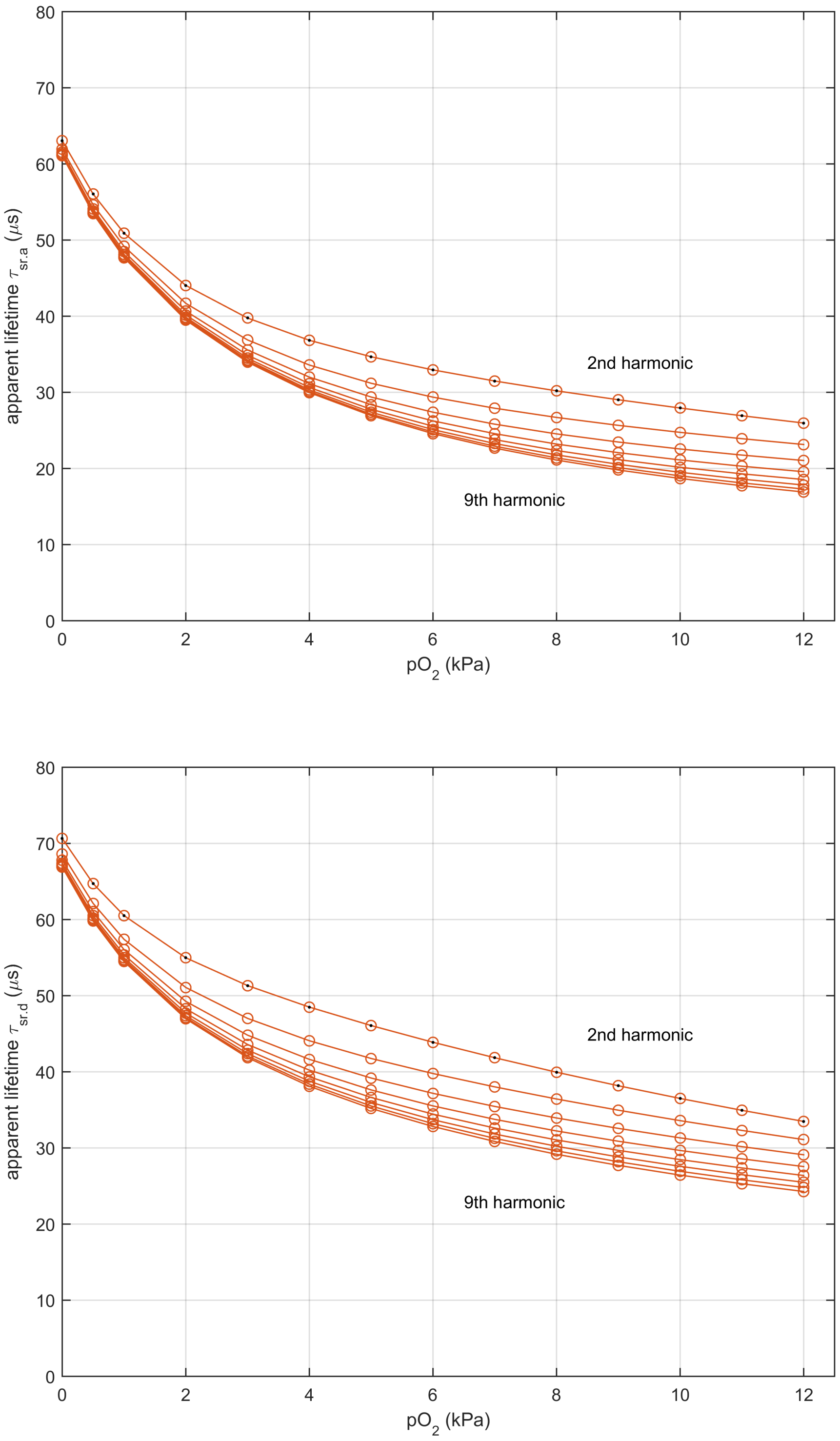


**Figure S15.** Estimated apparent lifetimes as a function of the concentration for the simulated two-sites model fitting the experimental data. Each line correspond to a harmonic referenced to the first one. Apparent lifetimes based on self-referenced normalized amplitudes (top panel) and delays (bottom panel).

## 7. Experimental lifetime estimations (conventional procedure)

### *7.1. Modulation factor and phase-shift measurements*

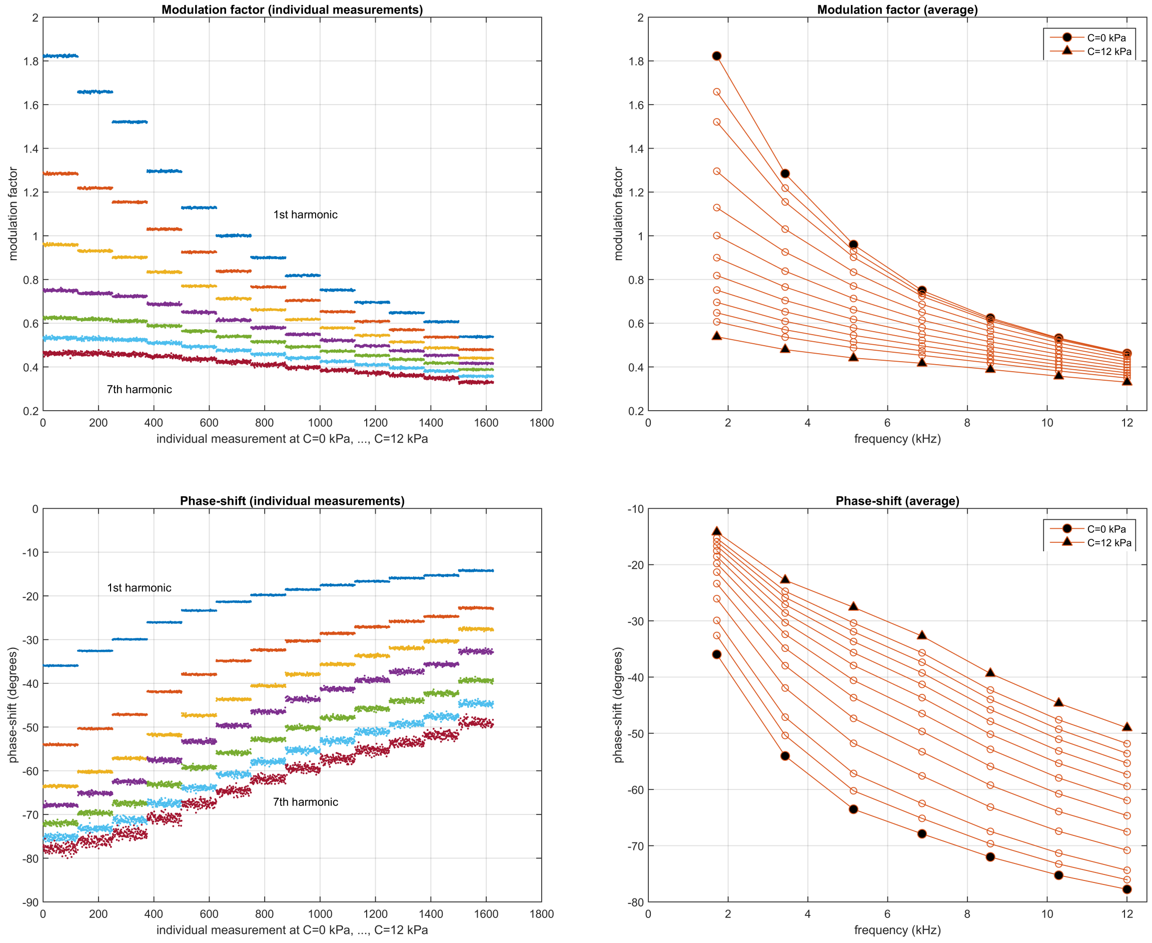


**Figure S16.** Modulation factor (top) and phase shift (bottom) measured in the calibration partition. The left panels show the individual measurements at increasing concentrations, from 0 to 12 kPa $pO_2$ (125 measurements for each concentration). The right panels show the average measurements.

| **Modulation factor $m(n)$ (arbitrary units) - Calibration partition** | | | | | | | |
|---|---|---|---|---|---|---|---|
| $C$ (kPa) | $f$=1715 Hz ($n$=1) | $f$=3430 Hz ($n$=2) | $f$=5145 Hz ($n$=3) | $f$=6860 Hz ($n$=4) | $f$=8575 Hz ($n$=5) | $f$=10290 Hz ($n$=6) | $f$=12005 Hz ($n$=7) |
| 0.00 | 1.8226 (0.0030) | 1.2841 (0.0028) | 0.9592 (0.0030) | 0.7499 (0.0036) | 0.6241 (0.0037) | 0.5321 (0.0046) | 0.4622 (0.0063) |
| 0.50 | 1.6586 (0.0029) | 1.2180 (0.0025) | 0.9306 (0.0026) | 0.7365 (0.0033) | 0.6177 (0.0033) | 0.5289 (0.0047) | 0.4609 (0.0060) |
| 1.00 | 1.5211 (0.0023) | 1.1544 (0.0022) | 0.9015 (0.0023) | 0.7231 (0.0030) | 0.6098 (0.0032) | 0.5239 (0.0038) | 0.4572 (0.0050) |
| 2.00 | 1.2955 (0.0026) | 1.0303 (0.0024) | 0.8340 (0.0026) | 0.6868 (0.0034) | 0.5878 (0.0030) | 0.5094 (0.0036) | 0.4477 (0.0046) |
| 3.00 | 1.1286 (0.0023) | 0.9254 (0.0022) | 0.7699 (0.0022) | 0.6500 (0.0032) | 0.5635 (0.0028) | 0.4928 (0.0032) | 0.4356 (0.0045) |
| 4.00 | 1.0006 (0.0027) | 0.8382 (0.0023) | 0.7126 (0.0025) | 0.6139 (0.0032) | 0.5391 (0.0026) | 0.4757 (0.0031) | 0.4230 (0.0047) |
| 5.00 | 0.8997 (0.0022) | 0.7653 (0.0020) | 0.6616 (0.0020) | 0.5795 (0.0030) | 0.5151 (0.0026) | 0.4581 (0.0032) | 0.4099 (0.0045) |
| 6.00 | 0.8183 (0.0022) | 0.7042 (0.0020) | 0.6172 (0.0020) | 0.5491 (0.0028) | 0.4926 (0.0024) | 0.4412 (0.0030) | 0.3972 (0.0041) |
| 7.00 | 0.7514 (0.0020) | 0.6525 (0.0019) | 0.5784 (0.0019) | 0.5212 (0.0027) | 0.4718 (0.0022) | 0.4249 (0.0026) | 0.3846 (0.0037) |
| 8.00 | 0.6955 (0.0021) | 0.6085 (0.0019) | 0.5444 (0.0020) | 0.4963 (0.0024) | 0.4523 (0.0024) | 0.4101 (0.0030) | 0.3729 (0.0041) |
| 9.00 | 0.6478 (0.0020) | 0.5699 (0.0020) | 0.5141 (0.0019) | 0.4736 (0.0024) | 0.4344 (0.0024) | 0.3956 (0.0028) | 0.3613 (0.0038) |
| 10.00 | 0.6067 (0.0019) | 0.5364 (0.0017) | 0.4873 (0.0020) | 0.4529 (0.0021) | 0.4176 (0.0022) | 0.3815 (0.0027) | 0.3491 (0.0044) |
| 12.00 | 0.5380 (0.0018) | 0.4792 (0.0017) | 0.4408 (0.0018) | 0.4167 (0.0021) | 0.3883 (0.0020) | 0.3575 (0.0024) | 0.3301 (0.0034) |

**Table S3.** Modulation factor (in arbitrary units) for each harmonic at different concentrations. Mean and standard deviation (in parentheses) for the calibration partition (estimated with 125 measurements at each concentration).

| **Modulation factor $m(n)$ (arbitrary units) - Evaluation partition** | | | | | | | |
|---|---|---|---|---|---|---|---|
| $C$ (kPa) | $f$=1715 Hz ($n$=1) | $f$=3430 Hz ($n$=2) | $f$=5145 Hz ($n$=3) | $f$=6860 Hz ($n$=4) | $f$=8575 Hz ($n$=5) | $f$=10290 Hz ($n$=6) | $f$=12005 Hz ($n$=7) |
| 0.00 | 1.8232 (0.0030) | 1.2848 (0.0027) | 0.9600 (0.0029) | 0.7505 (0.0034) | 0.6254 (0.0036) | 0.5325 (0.0041) | 0.4626 (0.0062) |
| 0.50 | 1.6583 (0.0026) | 1.2176 (0.0026) | 0.9303 (0.0029) | 0.7364 (0.0035) | 0.6169 (0.0036) | 0.5283 (0.0043) | 0.4601 (0.0059) |
| 1.00 | 1.5210 (0.0029) | 1.1544 (0.0028) | 0.9016 (0.0027) | 0.7231 (0.0034) | 0.6096 (0.0033) | 0.5241 (0.0035) | 0.4578 (0.0053) |
| 2.00 | 1.2956 (0.0024) | 1.0304 (0.0023) | 0.8339 (0.0025) | 0.6871 (0.0037) | 0.5876 (0.0030) | 0.5096 (0.0034) | 0.4475 (0.0047) |
| 3.00 | 1.1280 (0.0022) | 0.9250 (0.0023) | 0.7695 (0.0025) | 0.6489 (0.0034) | 0.5632 (0.0031) | 0.4925 (0.0037) | 0.4350 (0.0049) |
| 4.00 | 1.0005 (0.0023) | 0.8381 (0.0022) | 0.7124 (0.0023) | 0.6138 (0.0031) | 0.5391 (0.0028) | 0.4756 (0.0035) | 0.4233 (0.0046) |
| 5.00 | 0.8994 (0.0024) | 0.7650 (0.0021) | 0.6615 (0.0022) | 0.5799 (0.0029) | 0.5150 (0.0027) | 0.4578 (0.0031) | 0.4098 (0.0039) |
| 6.00 | 0.8183 (0.0021) | 0.7043 (0.0021) | 0.6173 (0.0023) | 0.5490 (0.0031) | 0.4928 (0.0027) | 0.4414 (0.0031) | 0.3973 (0.0039) |
| 7.00 | 0.7517 (0.0020) | 0.6528 (0.0017) | 0.5785 (0.0018) | 0.5214 (0.0028) | 0.4717 (0.0022) | 0.4254 (0.0028) | 0.3849 (0.0037) |
| 8.00 | 0.6952 (0.0021) | 0.6081 (0.0019) | 0.5442 (0.0021) | 0.4961 (0.0027) | 0.4523 (0.0024) | 0.4099 (0.0028) | 0.3727 (0.0038) |
| 9.00 | 0.6478 (0.0018) | 0.5700 (0.0019) | 0.5140 (0.0020) | 0.4733 (0.0027) | 0.4342 (0.0024) | 0.3952 (0.0031) | 0.3606 (0.0042) |
| 10.00 | 0.6066 (0.0022) | 0.5361 (0.0019) | 0.4872 (0.0020) | 0.4531 (0.0022) | 0.4180 (0.0021) | 0.3822 (0.0027) | 0.3506 (0.0039) |
| 12.00 | 0.5385 (0.0018) | 0.4796 (0.0018) | 0.4410 (0.0019) | 0.4170 (0.0024) | 0.3887 (0.0020) | 0.3580 (0.0026) | 0.3297 (0.0038) |

**Table S4.** Modulation factor (in arbitrary units) for each harmonic at different concentrations. Mean and standard deviation (in parentheses) for the evaluation partition (estimated with 125 measurements at each concentration).

| Phase shift $\phi(n)$ (degrees) - Calibration partition | | | | | | | |
|---|---|---|---|---|---|---|---|
| $C$ (kPa) | $f$=1715 Hz ($n$=1) | $f$=3430 Hz ($n$=2) | $f$=5145 Hz ($n$=3) | $f$=6860 Hz ($n$=4) | $f$=8575 Hz ($n$=5) | $f$=10290 Hz ($n$=6) | $f$=12005 Hz ($n$=7) |
| 0.00 | -35.954 (0.060) | -54.045 (0.111) | -63.513 (0.163) | -67.883 (0.243) | -72.008 (0.334) | -75.252 (0.477) | -77.768 (0.791) |
| 0.50 | -32.583 (0.051) | -50.372 (0.096) | -60.231 (0.154) | -65.134 (0.255) | -69.656 (0.302) | -73.263 (0.423) | -76.062 (0.723) |
| 1.00 | -29.928 (0.053) | -47.135 (0.093) | -57.153 (0.159) | -62.494 (0.275) | -67.451 (0.346) | -71.317 (0.526) | -74.379 (0.777) |
| 2.00 | -26.053 (0.055) | -41.924 (0.103) | -51.779 (0.171) | -57.576 (0.309) | -63.126 (0.344) | -67.438 (0.492) | -70.809 (0.765) |
| 3.00 | -23.353 (0.058) | -37.968 (0.105) | -47.328 (0.160) | -53.302 (0.293) | -59.241 (0.287) | -63.909 (0.381) | -67.519 (0.638) |
| 4.00 | -21.340 (0.060) | -34.861 (0.091) | -43.652 (0.143) | -49.675 (0.256) | -55.880 (0.263) | -60.778 (0.402) | -64.651 (0.604) |
| 5.00 | -19.787 (0.075) | -32.375 (0.110) | -40.574 (0.155) | -46.480 (0.263) | -52.855 (0.250) | -57.947 (0.376) | -61.958 (0.581) |
| 6.00 | -18.544 (0.073) | -30.298 (0.118) | -37.918 (0.180) | -43.647 (0.301) | -50.176 (0.290) | -55.340 (0.398) | -59.447 (0.602) |
| 7.00 | -17.528 (0.083) | -28.581 (0.120) | -35.654 (0.162) | -41.308 (0.279) | -47.875 (0.297) | -53.139 (0.438) | -57.339 (0.654) |
| 8.00 | -16.663 (0.071) | -27.097 (0.112) | -33.680 (0.183) | -39.241 (0.315) | -45.790 (0.314) | -51.079 (0.450) | -55.310 (0.647) |
| 9.00 | -15.929 (0.082) | -25.821 (0.118) | -31.936 (0.178) | -37.351 (0.310) | -43.987 (0.304) | -49.287 (0.419) | -53.592 (0.576) |
| 10.00 | -15.294 (0.080) | -24.714 (0.107) | -30.369 (0.175) | -35.668 (0.241) | -42.306 (0.304) | -47.607 (0.417) | -51.847 (0.622) |
| 12.00 | -14.218 (0.085) | -22.764 (0.116) | -27.584 (0.178) | -32.700 (0.296) | -39.357 (0.284) | -44.656 (0.406) | -49.032 (0.638) |

**Table S5.** Phase shift (in degrees) for each harmonic at different concentrations. Mean and standard deviation (in parentheses) for the calibration partition (estimated with 125 measurements at each concentration).

| Phase shift $\phi(n)$ (degrees) - Evaluation partition | | | | | | | |
|---|---|---|---|---|---|---|---|
| $C$ (kPa) | $f$=1715 Hz ($n$=1) | $f$=3430 Hz ($n$=2) | $f$=5145 Hz ($n$=3) | $f$=6860 Hz ($n$=4) | $f$=8575 Hz ($n$=5) | $f$=10290 Hz ($n$=6) | $f$=12005 Hz ($n$=7) |
| 0.00 | -35.942 (0.056) | -54.035 (0.102) | -63.510 (0.149) | -67.849 (0.249) | -72.087 (0.319) | -75.394 (0.536) | -77.941 (0.699) |
| 0.50 | -32.585 (0.058) | -50.375 (0.111) | -60.227 (0.161) | -65.157 (0.241) | -69.689 (0.292) | -73.271 (0.435) | -76.020 (0.703) |
| 1.00 | -29.926 (0.051) | -47.141 (0.102) | -57.173 (0.168) | -62.509 (0.274) | -67.426 (0.354) | -71.280 (0.531) | -74.291 (0.715) |
| 2.00 | -26.055 (0.053) | -41.933 (0.095) | -51.779 (0.154) | -57.578 (0.273) | -63.127 (0.283) | -67.424 (0.403) | -70.784 (0.654) |
| 3.00 | -23.365 (0.060) | -37.978 (0.093) | -47.342 (0.161) | -53.312 (0.320) | -59.287 (0.306) | -63.949 (0.434) | -67.600 (0.646) |
| 4.00 | -21.339 (0.067) | -34.864 (0.102) | -43.668 (0.151) | -49.652 (0.276) | -55.882 (0.300) | -60.792 (0.414) | -64.669 (0.656) |
| 5.00 | -19.791 (0.073) | -32.348 (0.113) | -40.541 (0.161) | -46.463 (0.315) | -52.862 (0.300) | -57.939 (0.429) | -61.931 (0.681) |
| 6.00 | -18.544 (0.080) | -30.307 (0.116) | -37.958 (0.147) | -43.763 (0.311) | -50.231 (0.284) | -55.418 (0.413) | -59.557 (0.606) |
| 7.00 | -17.527 (0.072) | -28.584 (0.114) | -35.667 (0.169) | -41.328 (0.311) | -47.840 (0.300) | -53.055 (0.404) | -57.273 (0.631) |
| 8.00 | -16.672 (0.085) | -27.118 (0.130) | -33.707 (0.204) | -39.292 (0.337) | -45.858 (0.340) | -51.164 (0.467) | -55.443 (0.676) |
| 9.00 | -15.922 (0.079) | -25.830 (0.119) | -31.931 (0.169) | -37.376 (0.314) | -43.950 (0.301) | -49.239 (0.448) | -53.545 (0.644) |
| 10.00 | -15.308 (0.075) | -24.707 (0.112) | -30.365 (0.191) | -35.678 (0.279) | -42.307 (0.298) | -47.602 (0.432) | -51.895 (0.702) |
| 12.00 | -14.225 (0.083) | -22.763 (0.116) | -27.583 (0.192) | -32.665 (0.313) | -39.379 (0.305) | -44.690 (0.413) | -48.919 (0.628) |

**Table S6.** Phase shift (in degrees) for each harmonic at different concentrations. Mean and standard deviation (in parentheses) for the evaluation partition (estimated with 125 measurements at each concentration).

## *7.2. Modulation factor and phase-shift based lifetime estimations*

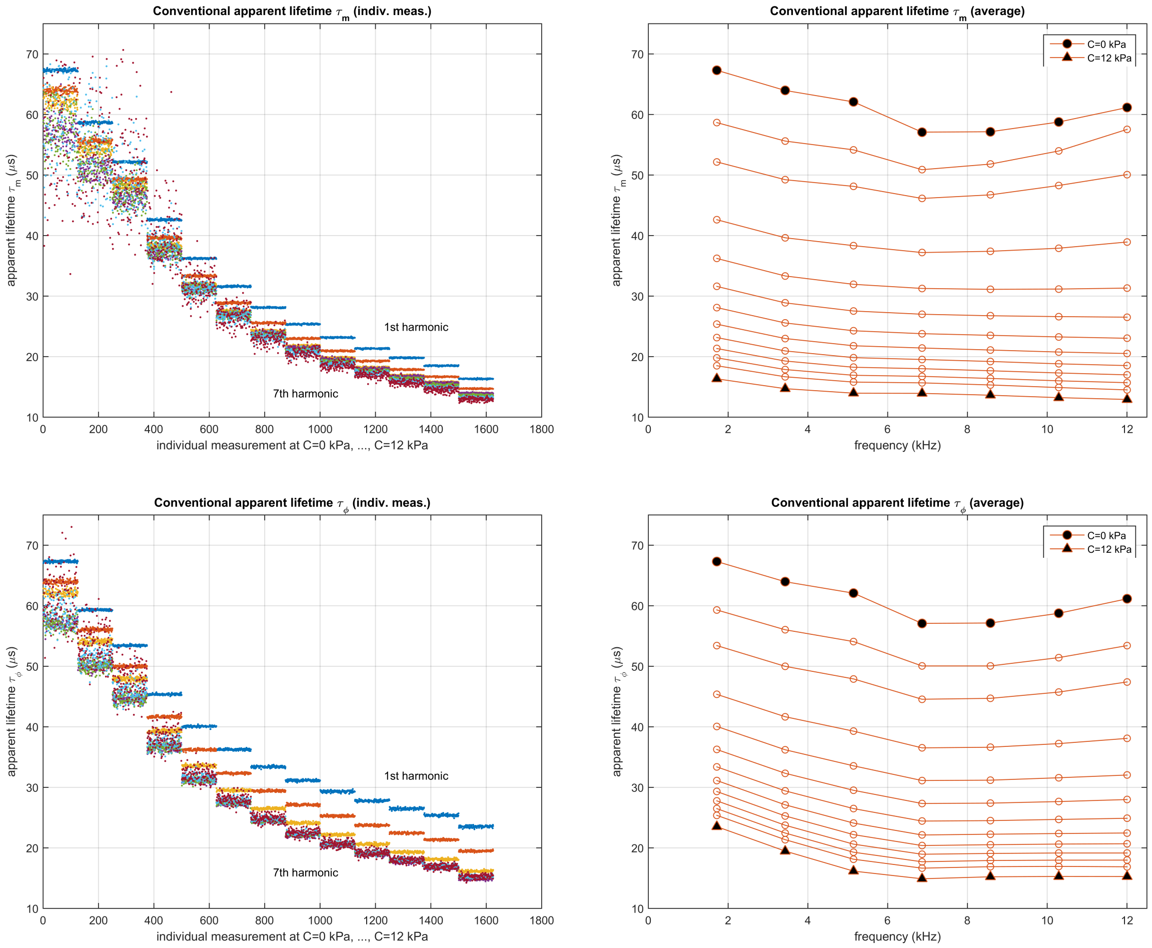


**Figure S17.** Modulation factor based lifetimes (top) and phase shift based lifetimes (bottom) estimated in the calibration partition. The left panels show the individual measurements at increasing concentrations, from 0 to 12 kPa $pO_2$ (125 measurements for each concentration). The right panels show the average estimations.

| Modulation factor based lifetime $\tau_m(n)$ ($\mu$s) - Calibration partition | | | | | | | |
|---|---|---|---|---|---|---|---|
| $C$ (kPa) | $f$=1715 Hz ($n$=1) | $f$=3430 Hz ($n$=2) | $f$=5145 Hz ($n$=3) | $f$=6860 Hz ($n$=4) | $f$=8575 Hz ($n$=5) | $f$=10290 Hz ($n$=6) | $f$=12005 Hz ($n$=7) |
| 0.00 | 67.310 (0.170) | 63.973 (0.399) | 62.099 (0.988) | 57.171 (1.970) | 57.287 (3.401) | 57.503 (5.650) | 54.461 (8.374) |
| 0.50 | 58.662 (0.142) | 55.610 (0.274) | 54.166 (0.618) | 50.920 (1.338) | 51.968 (2.580) | 54.036 (5.499) | 52.768 (7.339) |
| 1.00 | 52.167 (0.105) | 49.226 (0.203) | 48.128 (0.421) | 46.152 (0.969) | 46.811 (1.812) | 48.694 (3.999) | 50.220 (7.436) |
| 2.00 | 42.618 (0.104) | 39.632 (0.158) | 38.333 (0.307) | 37.205 (0.669) | 37.428 (0.982) | 38.027 (1.919) | 39.515 (4.486) |
| 3.00 | 36.219 (0.085) | 33.324 (0.122) | 31.943 (0.187) | 31.270 (0.432) | 31.126 (0.598) | 31.201 (1.000) | 31.506 (2.188) |
| 4.00 | 31.601 (0.093) | 28.876 (0.112) | 27.536 (0.170) | 27.005 (0.337) | 26.755 (0.396) | 26.629 (0.683) | 26.611 (1.476) |
| 5.00 | 28.105 (0.075) | 25.557 (0.087) | 24.271 (0.120) | 23.790 (0.250) | 23.523 (0.305) | 23.265 (0.523) | 23.079 (1.012) |
| 6.00 | 25.361 (0.075) | 22.988 (0.081) | 21.792 (0.108) | 21.424 (0.199) | 21.092 (0.236) | 20.762 (0.396) | 20.539 (0.717) |
| 7.00 | 23.153 (0.066) | 20.938 (0.073) | 19.829 (0.093) | 19.527 (0.176) | 19.200 (0.190) | 18.811 (0.288) | 18.533 (0.532) |
| 8.00 | 21.335 (0.067) | 19.272 (0.072) | 18.242 (0.091) | 18.005 (0.141) | 17.664 (0.176) | 17.302 (0.280) | 17.001 (0.489) |
| 9.00 | 19.801 (0.063) | 17.863 (0.071) | 16.914 (0.081) | 16.742 (0.131) | 16.395 (0.161) | 16.005 (0.237) | 15.702 (0.406) |
| 10.00 | 18.495 (0.061) | 16.670 (0.060) | 15.793 (0.082) | 15.665 (0.104) | 15.316 (0.137) | 14.888 (0.200) | 14.512 (0.401) |
| 12.00 | 16.331 (0.057) | 14.705 (0.057) | 13.958 (0.069) | 13.930 (0.096) | 13.624 (0.106) | 13.222 (0.154) | 12.925 (0.259) |

**Table S7.** Modulation factor based lifetime ($\mu$s) for each harmonic at different concentrations. Mean and standard deviation (in parentheses) for the calibration partition (estimated with 125 measurements at each concentration).

| Modulation factor based lifetime $\tau_m(n)$ ($\mu$s) - Evaluation partition | | | | | | | |
|---|---|---|---|---|---|---|---|
| $C$ (kPa) | $f$=1715 Hz ($n$=1) | $f$=3430 Hz ($n$=2) | $f$=5145 Hz ($n$=3) | $f$=6860 Hz ($n$=4) | $f$=8575 Hz ($n$=5) | $f$=10290 Hz ($n$=6) | $f$=12005 Hz ($n$=7) |
| 0.00 | 67.282 (0.171) | 63.950 (0.395) | 62.089 (0.934) | 57.066 (1.845) | 57.614 (3.356) | 58.101 (5.167) | 54.977 (8.024) |
| 0.50 | 58.596 (0.128) | 55.476 (0.286) | 53.930 (0.690) | 50.583 (1.393) | 50.622 (2.543) | 53.782 (5.636) | 53.823 (8.533) |
| 1.00 | 52.121 (0.132) | 49.165 (0.250) | 48.016 (0.496) | 45.910 (1.072) | 46.128 (1.780) | 48.631 (3.544) | 52.084 (8.921) |
| 2.00 | 42.590 (0.097) | 39.596 (0.149) | 38.248 (0.292) | 37.123 (0.702) | 37.067 (0.944) | 38.031 (1.771) | 39.315 (4.425) |
| 3.00 | 36.169 (0.082) | 33.268 (0.126) | 31.856 (0.213) | 31.032 (0.457) | 30.860 (0.629) | 31.086 (1.162) | 31.256 (2.299) |
| 4.00 | 31.575 (0.082) | 28.847 (0.106) | 27.488 (0.160) | 26.921 (0.315) | 26.615 (0.421) | 26.608 (0.787) | 26.685 (1.481) |
| 5.00 | 28.075 (0.081) | 25.520 (0.090) | 24.240 (0.129) | 23.776 (0.241) | 23.409 (0.315) | 23.199 (0.512) | 23.026 (0.875) |
| 6.00 | 25.342 (0.071) | 22.972 (0.086) | 21.769 (0.119) | 21.371 (0.222) | 21.033 (0.260) | 20.782 (0.407) | 20.538 (0.692) |
| 7.00 | 23.146 (0.066) | 20.932 (0.067) | 19.813 (0.089) | 19.507 (0.178) | 19.124 (0.185) | 18.852 (0.313) | 18.569 (0.533) |
| 8.00 | 21.310 (0.067) | 19.242 (0.072) | 18.212 (0.094) | 17.967 (0.157) | 17.602 (0.178) | 17.269 (0.270) | 16.970 (0.454) |
| 9.00 | 19.791 (0.059) | 17.851 (0.067) | 16.892 (0.087) | 16.696 (0.145) | 16.329 (0.161) | 15.968 (0.255) | 15.632 (0.432) |
| 10.00 | 18.479 (0.069) | 16.649 (0.065) | 15.773 (0.081) | 15.647 (0.113) | 15.292 (0.128) | 14.930 (0.201) | 14.649 (0.367) |
| 12.00 | 16.336 (0.057) | 14.706 (0.062) | 13.953 (0.074) | 13.927 (0.108) | 13.612 (0.106) | 13.250 (0.167) | 12.895 (0.286) |

**Table S8.** Modulation factor based lifetime ($\mu$s) for each harmonic at different concentrations. Mean and standard deviation (in parentheses) for the evaluation partition (estimated with 125 measurements at each concentration).

| **Phase shift based lifetime $\tau_\phi(n)$ ($\mu$s) - Calibration partition** | | | | | | | |
|---|---|---|---|---|---|---|---|
| C (kPa) | $f$=1715 Hz ($n$=1) | $f$=3430 Hz ($n$=2) | $f$=5145 Hz ($n$=3) | $f$=6860 Hz ($n$=4) | $f$=8575 Hz ($n$=5) | $f$=10290 Hz ($n$=6) | $f$=12005 Hz ($n$=7) |
| 0.00 | 67.310 (0.148) | 63.971 (0.261) | 62.083 (0.444) | 57.093 (0.692) | 57.171 (1.138) | 58.818 (1.996) | 61.412 (4.151) |
| 0.50 | 59.310 (0.117) | 56.034 (0.191) | 54.083 (0.338) | 50.065 (0.584) | 50.068 (0.812) | 51.467 (1.378) | 53.566 (2.934) |
| 1.00 | 53.424 (0.114) | 49.996 (0.164) | 47.915 (0.292) | 44.561 (0.522) | 44.712 (0.765) | 45.777 (1.404) | 47.535 (2.469) |
| 2.00 | 45.368 (0.110) | 41.668 (0.151) | 39.281 (0.241) | 36.527 (0.436) | 36.633 (0.548) | 37.245 (0.904) | 38.153 (1.643) |
| 3.00 | 40.068 (0.112) | 36.211 (0.137) | 33.556 (0.188) | 31.131 (0.332) | 31.189 (0.355) | 31.591 (0.534) | 32.063 (1.017) |
| 4.00 | 36.256 (0.112) | 32.323 (0.110) | 29.512 (0.147) | 27.334 (0.248) | 27.395 (0.271) | 27.656 (0.455) | 28.001 (0.762) |
| 5.00 | 33.388 (0.137) | 29.418 (0.125) | 26.490 (0.145) | 24.432 (0.225) | 24.502 (0.222) | 24.705 (0.360) | 24.901 (0.607) |
| 6.00 | 31.130 (0.131) | 27.112 (0.129) | 24.097 (0.156) | 22.131 (0.232) | 22.259 (0.229) | 22.373 (0.332) | 22.469 (0.539) |
| 7.00 | 29.311 (0.147) | 25.279 (0.127) | 22.191 (0.133) | 20.389 (0.200) | 20.524 (0.214) | 20.632 (0.329) | 20.690 (0.522) |
| 8.00 | 27.776 (0.126) | 23.741 (0.114) | 20.615 (0.143) | 18.950 (0.212) | 19.081 (0.209) | 19.157 (0.308) | 19.161 (0.463) |
| 9.00 | 26.486 (0.144) | 22.452 (0.118) | 19.282 (0.133) | 17.707 (0.198) | 17.917 (0.190) | 17.976 (0.266) | 17.982 (0.376) |
| 10.00 | 25.378 (0.139) | 21.355 (0.105) | 18.126 (0.127) | 16.652 (0.148) | 16.893 (0.180) | 16.945 (0.248) | 16.881 (0.378) |
| 12.00 | 23.514 (0.147) | 19.470 (0.111) | 16.161 (0.123) | 14.895 (0.169) | 15.223 (0.154) | 15.284 (0.216) | 15.273 (0.342) |

**Table S9.** Phase shift based lifetime ($\mu$s) for each harmonic at different concentrations. Mean and standard deviation (in parentheses) for the calibration partition (estimated with 125 measurements at each concentration).

| **Phase shift based lifetime $\tau_\phi(n)$ ($\mu$s) - Evaluation partition** | | | | | | | |
|---|---|---|---|---|---|---|---|
| C (kPa) | $f$=1715 Hz ($n$=1) | $f$=3430 Hz ($n$=2) | $f$=5145 Hz ($n$=3) | $f$=6860 Hz ($n$=4) | $f$=8575 Hz ($n$=5) | $f$=10290 Hz ($n$=6) | $f$=12005 Hz ($n$=7) |
| 0.00 | 67.282 (0.139) | 63.948 (0.239) | 62.074 (0.405) | 56.997 (0.708) | 57.438 (1.086) | 59.435 (2.280) | 62.265 (3.732) |
| 0.50 | 59.314 (0.133) | 56.040 (0.220) | 54.075 (0.352) | 50.117 (0.555) | 50.156 (0.790) | 51.496 (1.424) | 53.386 (2.776) |
| 1.00 | 53.420 (0.110) | 50.006 (0.179) | 47.951 (0.309) | 44.590 (0.521) | 44.658 (0.780) | 45.681 (1.389) | 47.236 (2.282) |
| 2.00 | 45.372 (0.106) | 41.681 (0.139) | 39.281 (0.217) | 36.529 (0.386) | 36.631 (0.448) | 37.212 (0.735) | 38.082 (1.414) |
| 3.00 | 40.092 (0.114) | 36.224 (0.121) | 33.572 (0.189) | 31.142 (0.363) | 31.247 (0.381) | 31.649 (0.608) | 32.193 (1.026) |
| 4.00 | 36.255 (0.126) | 32.326 (0.123) | 29.529 (0.155) | 27.312 (0.266) | 27.397 (0.309) | 27.672 (0.469) | 28.027 (0.831) |
| 5.00 | 33.395 (0.133) | 29.388 (0.129) | 26.458 (0.150) | 24.418 (0.270) | 24.510 (0.266) | 24.699 (0.411) | 24.877 (0.713) |
| 6.00 | 31.131 (0.143) | 27.122 (0.126) | 24.132 (0.127) | 22.221 (0.242) | 22.302 (0.225) | 22.439 (0.347) | 22.567 (0.547) |
| 7.00 | 29.308 (0.129) | 25.282 (0.119) | 22.202 (0.138) | 20.403 (0.224) | 20.499 (0.215) | 20.569 (0.301) | 20.638 (0.500) |
| 8.00 | 27.792 (0.150) | 23.764 (0.133) | 20.636 (0.159) | 18.985 (0.228) | 19.126 (0.227) | 19.216 (0.321) | 19.257 (0.488) |
| 9.00 | 26.475 (0.138) | 22.461 (0.119) | 19.278 (0.127) | 17.723 (0.201) | 17.893 (0.188) | 17.946 (0.285) | 17.952 (0.425) |
| 10.00 | 25.402 (0.131) | 21.349 (0.109) | 18.123 (0.139) | 16.658 (0.171) | 16.894 (0.177) | 16.942 (0.256) | 16.912 (0.426) |
| 12.00 | 23.525 (0.143) | 19.470 (0.110) | 16.160 (0.132) | 14.875 (0.179) | 15.235 (0.165) | 15.302 (0.221) | 15.211 (0.337) |

**Table S10.** Phase shift based lifetime ($\mu$s) for each harmonic at different concentrations. Mean and standard deviation (in parentheses) for the evaluation partition (estimated with 125 measurements at each concentration).

## 8. Self-referenced amplitudes

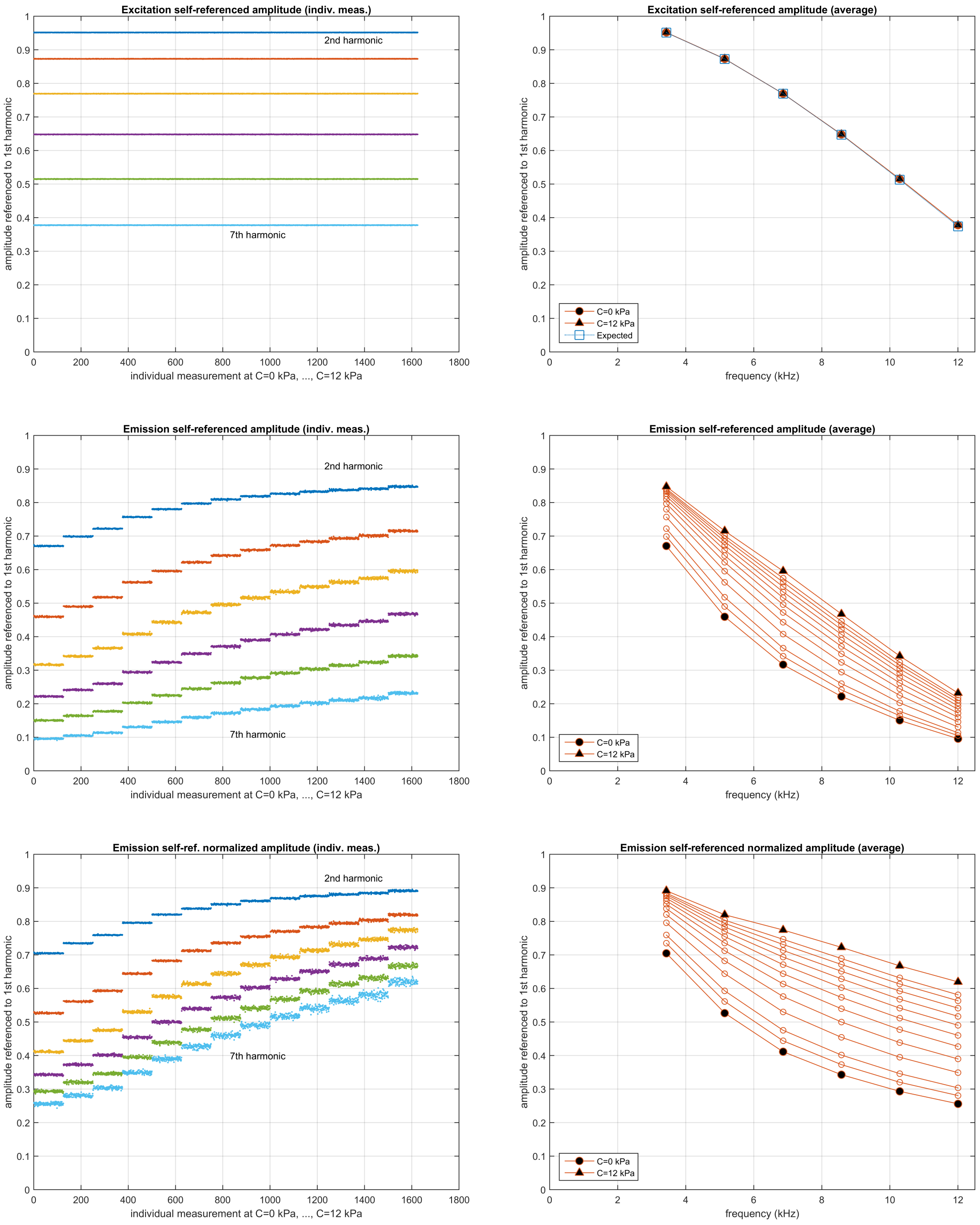


**Figure S18.** Self-referenced amplitude of the excitation signal (top); self-referenced amplitude of the emission signal (center); self-referenced normalized amplitude of the emission signal (bottom), for the calibration partition. First harmonic used as reference. The self-referenced normalized amplitudes for the emission signals have been calculated using the expected value of the self-referenced amplitude in the excitation signal. The left panels show the individual measurements at increasing concentrations, from 0 to 12 kPa $pO_2$ (125 measurements for each concentration). The right panels show the average estimations.

| **Self-referenced amplitudes for the excitation signal $A_{exc}(n)/A_{exc}(1)$ - Calibration partition** | | | | | | |
|---|---|---|---|---|---|---|
| $C$ (kPa) | $f$=3430 Hz ($n$=2) | $f$=5145 Hz ($n$=3) | $f$=6860 Hz ($n$=4) | $f$=8575 Hz ($n$=5) | $f$=10290 Hz ($n$=6) | $f$=12005 Hz ($n$=7) |
| 0.00 | 0.95146 (0.00014) | 0.87300 (0.00020) | 0.76921 (0.00021) | 0.64794 (0.00025) | 0.51506 (0.00027) | 0.37758 (0.00029) |
| 0.50 | 0.95147 (0.00015) | 0.87303 (0.00018) | 0.76921 (0.00019) | 0.64794 (0.00023) | 0.51506 (0.00028) | 0.37760 (0.00029) |
| 1.00 | 0.95147 (0.00016) | 0.87305 (0.00017) | 0.76926 (0.00021) | 0.64799 (0.00023) | 0.51512 (0.00026) | 0.37763 (0.00025) |
| 2.00 | 0.95150 (0.00015) | 0.87310 (0.00017) | 0.76930 (0.00020) | 0.64802 (0.00022) | 0.51513 (0.00025) | 0.37765 (0.00027) |
| 3.00 | 0.95151 (0.00016) | 0.87311 (0.00016) | 0.76932 (0.00019) | 0.64803 (0.00023) | 0.51513 (0.00026) | 0.37764 (0.00026) |
| 4.00 | 0.95150 (0.00015) | 0.87309 (0.00016) | 0.76931 (0.00020) | 0.64801 (0.00022) | 0.51510 (0.00022) | 0.37762 (0.00023) |
| 5.00 | 0.95147 (0.00016) | 0.87310 (0.00017) | 0.76930 (0.00021) | 0.64801 (0.00024) | 0.51511 (0.00023) | 0.37761 (0.00024) |
| 6.00 | 0.95148 (0.00016) | 0.87308 (0.00017) | 0.76931 (0.00020) | 0.64800 (0.00025) | 0.51508 (0.00027) | 0.37759 (0.00027) |
| 7.00 | 0.95152 (0.00017) | 0.87313 (0.00017) | 0.76935 (0.00021) | 0.64806 (0.00023) | 0.51516 (0.00025) | 0.37763 (0.00025) |
| 8.00 | 0.95147 (0.00016) | 0.87311 (0.00017) | 0.76931 (0.00023) | 0.64798 (0.00025) | 0.51508 (0.00027) | 0.37758 (0.00028) |
| 9.00 | 0.95148 (0.00016) | 0.87312 (0.00017) | 0.76934 (0.00021) | 0.64803 (0.00023) | 0.51512 (0.00025) | 0.37764 (0.00027) |
| 10.00 | 0.95151 (0.00016) | 0.87314 (0.00018) | 0.76937 (0.00020) | 0.64805 (0.00024) | 0.51514 (0.00026) | 0.37764 (0.00026) |
| 12.00 | 0.95149 (0.00017) | 0.87309 (0.00019) | 0.76931 (0.00021) | 0.64801 (0.00027) | 0.51510 (0.00028) | 0.37760 (0.00030) |
| Expected | 0.95106 | 0.87268 | 0.76942 | 0.64721 | 0.51295 | 0.37400 |

**Table S11.** Self-referenced amplitudes for the excitation signal $A_{exc}(n)/A_{exc}(1)$ for each harmonic at different concentrations. Mean and standard deviation (in parentheses) for the calibration partition (estimated with 125 measurements at each concentration).

| **Self-referenced amplitudes for the excitation signal $A_{exc}(n)/A_{exc}(1)$ - Evaluation partition** | | | | | | |
|---|---|---|---|---|---|---|
| $C$ (kPa) | $f$=3430 Hz ($n$=2) | $f$=5145 Hz ($n$=3) | $f$=6860 Hz ($n$=4) | $f$=8575 Hz ($n$=5) | $f$=10290 Hz ($n$=6) | $f$=12005 Hz ($n$=7) |
| 0.00 | 0.95146 (0.00016) | 0.87301 (0.00017) | 0.76920 (0.00023) | 0.64793 (0.00028) | 0.51506 (0.00032) | 0.37759 (0.00033) |
| 0.50 | 0.95148 (0.00016) | 0.87302 (0.00018) | 0.76923 (0.00023) | 0.64794 (0.00027) | 0.51506 (0.00031) | 0.37756 (0.00032) |
| 1.00 | 0.95148 (0.00014) | 0.87305 (0.00018) | 0.76923 (0.00021) | 0.64796 (0.00026) | 0.51508 (0.00029) | 0.37759 (0.00028) |
| 2.00 | 0.95150 (0.00016) | 0.87310 (0.00017) | 0.76927 (0.00023) | 0.64799 (0.00025) | 0.51509 (0.00030) | 0.37761 (0.00030) |
| 3.00 | 0.95148 (0.00015) | 0.87309 (0.00015) | 0.76932 (0.00019) | 0.64802 (0.00024) | 0.51512 (0.00026) | 0.37764 (0.00026) |
| 4.00 | 0.95149 (0.00016) | 0.87306 (0.00018) | 0.76927 (0.00023) | 0.64795 (0.00028) | 0.51505 (0.00029) | 0.37756 (0.00030) |
| 5.00 | 0.95150 (0.00015) | 0.87310 (0.00019) | 0.76930 (0.00022) | 0.64800 (0.00026) | 0.51508 (0.00028) | 0.37758 (0.00029) |
| 6.00 | 0.95149 (0.00015) | 0.87309 (0.00019) | 0.76930 (0.00021) | 0.64801 (0.00025) | 0.51509 (0.00027) | 0.37759 (0.00027) |
| 7.00 | 0.95149 (0.00016) | 0.87312 (0.00017) | 0.76933 (0.00020) | 0.64801 (0.00025) | 0.51511 (0.00028) | 0.37762 (0.00028) |
| 8.00 | 0.95149 (0.00016) | 0.87309 (0.00017) | 0.76930 (0.00020) | 0.64798 (0.00023) | 0.51506 (0.00028) | 0.37753 (0.00027) |
| 9.00 | 0.95149 (0.00015) | 0.87311 (0.00016) | 0.76933 (0.00020) | 0.64803 (0.00026) | 0.51510 (0.00028) | 0.37757 (0.00028) |
| 10.00 | 0.95148 (0.00017) | 0.87311 (0.00017) | 0.76935 (0.00021) | 0.64803 (0.00025) | 0.51513 (0.00028) | 0.37761 (0.00027) |
| 12.00 | 0.95150 (0.00017) | 0.87312 (0.00016) | 0.76932 (0.00021) | 0.64802 (0.00025) | 0.51509 (0.00029) | 0.37760 (0.00029) |
| Expected | 0.95106 | 0.87268 | 0.76942 | 0.64721 | 0.51295 | 0.37400 |

**Table S12.** Self-referenced amplitudes for the excitation signal $A_{exc}(n)/A_{exc}(1)$ for each harmonic at different concentrations. Mean and standard deviation (in parentheses) for the evaluation partition (estimated with 125 measurements at each concentration).

| **Self-referenced amplitudes for the emission signal $A_{em}(n)/A_{em}(1)$ - Calibration partition** | | | | | | |
|---|---|---|---|---|---|---|
| C (kPa) | $f$=3430 Hz ($n$=2) | $f$=5145 Hz ($n$=3) | $f$=6860 Hz ($n$=4) | $f$=8575 Hz ($n$=5) | $f$=10290 Hz ($n$=6) | $f$=12005 Hz ($n$=7) |
| 0.00 | 0.6704 (0.0010) | 0.4595 (0.0013) | 0.3165 (0.0014) | 0.2219 (0.0012) | 0.1504 (0.0012) | 0.0958 (0.0013) |
| 0.50 | 0.6987 (0.0009) | 0.4898 (0.0012) | 0.3416 (0.0015) | 0.2413 (0.0013) | 0.1642 (0.0014) | 0.1049 (0.0013) |
| 1.00 | 0.7221 (0.0008) | 0.5174 (0.0012) | 0.3657 (0.0015) | 0.2598 (0.0014) | 0.1774 (0.0013) | 0.1135 (0.0013) |
| 2.00 | 0.7567 (0.0008) | 0.5621 (0.0013) | 0.4078 (0.0020) | 0.2940 (0.0014) | 0.2026 (0.0013) | 0.1305 (0.0013) |
| 3.00 | 0.7801 (0.0008) | 0.5956 (0.0011) | 0.4431 (0.0021) | 0.3236 (0.0016) | 0.2249 (0.0015) | 0.1457 (0.0015) |
| 4.00 | 0.7970 (0.0010) | 0.6218 (0.0014) | 0.4720 (0.0022) | 0.3491 (0.0016) | 0.2449 (0.0016) | 0.1597 (0.0017) |
| 5.00 | 0.8093 (0.0011) | 0.6420 (0.0014) | 0.4955 (0.0023) | 0.3710 (0.0017) | 0.2623 (0.0018) | 0.1720 (0.0019) |
| 6.00 | 0.8189 (0.0012) | 0.6586 (0.0015) | 0.5162 (0.0025) | 0.3901 (0.0019) | 0.2777 (0.0019) | 0.1833 (0.0019) |
| 7.00 | 0.8262 (0.0012) | 0.6721 (0.0015) | 0.5336 (0.0024) | 0.4069 (0.0018) | 0.2913 (0.0019) | 0.1933 (0.0019) |
| 8.00 | 0.8324 (0.0012) | 0.6834 (0.0016) | 0.5489 (0.0023) | 0.4214 (0.0019) | 0.3037 (0.0021) | 0.2024 (0.0022) |
| 9.00 | 0.8372 (0.0015) | 0.6930 (0.0017) | 0.5625 (0.0027) | 0.4346 (0.0021) | 0.3146 (0.0020) | 0.2106 (0.0021) |
| 10.00 | 0.8411 (0.0014) | 0.7013 (0.0020) | 0.5744 (0.0022) | 0.4461 (0.0020) | 0.3239 (0.0021) | 0.2173 (0.0027) |
| 12.00 | 0.8476 (0.0016) | 0.7154 (0.0018) | 0.5958 (0.0026) | 0.4677 (0.0022) | 0.3423 (0.0022) | 0.2317 (0.0023) |

**Table S13.** Self-referenced amplitudes for the emission signal $A_{em}(n)/A_{em}(1)$ for each harmonic at different concentrations. Mean and standard deviation (in parentheses) for the calibration partition (estimated with 125 measurements at each concentration).

| **Self-referenced amplitudes for the emission signal $A_{em}(n)/A_{em}(1)$ - Evaluation partition** | | | | | | |
|---|---|---|---|---|---|---|
| C (kPa) | $f$=3430 Hz ($n$=2) | $f$=5145 Hz ($n$=3) | $f$=6860 Hz ($n$=4) | $f$=8575 Hz ($n$=5) | $f$=10290 Hz ($n$=6) | $f$=12005 Hz ($n$=7) |
| 0.00 | 0.6705 (0.0009) | 0.4597 (0.0011) | 0.3166 (0.0014) | 0.2223 (0.0013) | 0.1504 (0.0012) | 0.0958 (0.0013) |
| 0.50 | 0.6986 (0.0009) | 0.4898 (0.0013) | 0.3416 (0.0015) | 0.2410 (0.0014) | 0.1641 (0.0013) | 0.1047 (0.0013) |
| 1.00 | 0.7222 (0.0009) | 0.5175 (0.0013) | 0.3657 (0.0017) | 0.2597 (0.0013) | 0.1775 (0.0012) | 0.1136 (0.0013) |
| 2.00 | 0.7568 (0.0009) | 0.5620 (0.0012) | 0.4080 (0.0019) | 0.2939 (0.0014) | 0.2026 (0.0013) | 0.1304 (0.0014) |
| 3.00 | 0.7802 (0.0010) | 0.5956 (0.0014) | 0.4426 (0.0022) | 0.3236 (0.0018) | 0.2249 (0.0017) | 0.1456 (0.0017) |
| 4.00 | 0.7971 (0.0010) | 0.6217 (0.0014) | 0.4719 (0.0021) | 0.3491 (0.0016) | 0.2449 (0.0017) | 0.1597 (0.0017) |
| 5.00 | 0.8093 (0.0011) | 0.6422 (0.0015) | 0.4960 (0.0021) | 0.3710 (0.0017) | 0.2622 (0.0016) | 0.1720 (0.0016) |
| 6.00 | 0.8190 (0.0012) | 0.6586 (0.0015) | 0.5161 (0.0025) | 0.3903 (0.0019) | 0.2779 (0.0018) | 0.1833 (0.0017) |
| 7.00 | 0.8263 (0.0012) | 0.6719 (0.0015) | 0.5337 (0.0026) | 0.4066 (0.0019) | 0.2915 (0.0020) | 0.1933 (0.0020) |
| 8.00 | 0.8323 (0.0013) | 0.6834 (0.0016) | 0.5490 (0.0026) | 0.4216 (0.0019) | 0.3037 (0.0020) | 0.2024 (0.0019) |
| 9.00 | 0.8371 (0.0017) | 0.6928 (0.0020) | 0.5620 (0.0027) | 0.4343 (0.0021) | 0.3143 (0.0023) | 0.2102 (0.0024) |
| 10.00 | 0.8409 (0.0013) | 0.7013 (0.0019) | 0.5746 (0.0024) | 0.4465 (0.0019) | 0.3245 (0.0020) | 0.2183 (0.0024) |
| 12.00 | 0.8474 (0.0017) | 0.7150 (0.0021) | 0.5958 (0.0030) | 0.4678 (0.0021) | 0.3424 (0.0024) | 0.2312 (0.0027) |

**Table S14.** Self-referenced amplitudes for the emission signal $A_{em}(n)/A_{em}(1)$ for each harmonic at different concentrations. Mean and standard deviation (in parentheses) for the evaluation partition (estimated with 125 measurements at each concentration).

| **Self-referenced normalized amplitudes for the emission signal** $[A_{em}(n)/A_{em}(1)]/[\text{sinc}(n/10)/\text{sinc}(1/10)]$ **Calibration partition** | | | | | | |
|---|---|---|---|---|---|---|
| $C$ (kPa) | $f$=3430 Hz ($n$=2) | $f$=5145 Hz ($n$=3) | $f$=6860 Hz ($n$=4) | $f$=8575 Hz ($n$=5) | $f$=10290 Hz ($n$=6) | $f$=12005 Hz ($n$=7) |
| 0.00 | 0.7049 (0.0010) | 0.5265 (0.0015) | 0.4113 (0.0019) | 0.3428 (0.0019) | 0.2931 (0.0024) | 0.2560 (0.0035) |
| 0.50 | 0.7347 (0.0009) | 0.5613 (0.0013) | 0.4439 (0.0019) | 0.3729 (0.0020) | 0.3202 (0.0028) | 0.2806 (0.0036) |
| 1.00 | 0.7592 (0.0009) | 0.5929 (0.0014) | 0.4753 (0.0020) | 0.4014 (0.0022) | 0.3459 (0.0026) | 0.3035 (0.0034) |
| 2.00 | 0.7957 (0.0009) | 0.6441 (0.0015) | 0.5300 (0.0026) | 0.4543 (0.0022) | 0.3949 (0.0026) | 0.3490 (0.0035) |
| 3.00 | 0.8203 (0.0009) | 0.6825 (0.0013) | 0.5759 (0.0027) | 0.4999 (0.0025) | 0.4385 (0.0029) | 0.3897 (0.0040) |
| 4.00 | 0.8380 (0.0010) | 0.7125 (0.0016) | 0.6135 (0.0029) | 0.5394 (0.0025) | 0.4774 (0.0031) | 0.4269 (0.0047) |
| 5.00 | 0.8510 (0.0012) | 0.7356 (0.0017) | 0.6440 (0.0030) | 0.5732 (0.0027) | 0.5113 (0.0035) | 0.4600 (0.0051) |
| 6.00 | 0.8610 (0.0012) | 0.7546 (0.0017) | 0.6710 (0.0032) | 0.6027 (0.0029) | 0.5414 (0.0037) | 0.4901 (0.0050) |
| 7.00 | 0.8688 (0.0012) | 0.7701 (0.0017) | 0.6936 (0.0032) | 0.6287 (0.0027) | 0.5680 (0.0036) | 0.5168 (0.0051) |
| 8.00 | 0.8753 (0.0013) | 0.7831 (0.0018) | 0.7134 (0.0030) | 0.6512 (0.0030) | 0.5922 (0.0042) | 0.5413 (0.0059) |
| 9.00 | 0.8802 (0.0015) | 0.7941 (0.0020) | 0.7311 (0.0036) | 0.6715 (0.0032) | 0.6133 (0.0039) | 0.5632 (0.0056) |
| 10.00 | 0.8844 (0.0015) | 0.8036 (0.0023) | 0.7465 (0.0029) | 0.6892 (0.0031) | 0.6315 (0.0042) | 0.5810 (0.0072) |
| 12.00 | 0.8912 (0.0017) | 0.8197 (0.0021) | 0.7743 (0.0034) | 0.7226 (0.0034) | 0.6673 (0.0043) | 0.6195 (0.0062) |

**Table S15.** Self-referenced normalized amplitudes for the emission signal $[A_{em}(n)/A_{em}(1)]/[\text{sinc(n/10)/sinc(1/10)}]$ for each harmonic at different concentrations. Mean and standard deviation (in parentheses) for the calibration partition (estimated with 125 measurements at each concentration).

| **Self-referenced normalized amplitudes for the emission signal** $[A_{em}(n)/A_{em}(1)]/[\text{sinc}(n/10)/\text{sinc}(1/10)]$ **Evaluation partition** | | | | | | |
|---|---|---|---|---|---|---|
| $C$ (kPa) | $f$=3430 Hz ($n$=2) | $f$=5145 Hz ($n$=3) | $f$=6860 Hz ($n$=4) | $f$=8575 Hz ($n$=5) | $f$=10290 Hz ($n$=6) | $f$=12005 Hz ($n$=7) |
| 0.00 | 0.7050 (0.0009) | 0.5267 (0.0013) | 0.4115 (0.0018) | 0.3434 (0.0020) | 0.2933 (0.0023) | 0.2561 (0.0034) |
| 0.50 | 0.7346 (0.0010) | 0.5612 (0.0015) | 0.4439 (0.0020) | 0.3724 (0.0021) | 0.3199 (0.0025) | 0.2801 (0.0036) |
| 1.00 | 0.7593 (0.0010) | 0.5930 (0.0014) | 0.4753 (0.0022) | 0.4013 (0.0021) | 0.3460 (0.0022) | 0.3038 (0.0035) |
| 2.00 | 0.7957 (0.0009) | 0.6440 (0.0014) | 0.5302 (0.0024) | 0.4541 (0.0021) | 0.3949 (0.0025) | 0.3487 (0.0037) |
| 3.00 | 0.8204 (0.0010) | 0.6825 (0.0016) | 0.5752 (0.0028) | 0.4999 (0.0027) | 0.4385 (0.0033) | 0.3894 (0.0044) |
| 4.00 | 0.8381 (0.0011) | 0.7124 (0.0016) | 0.6133 (0.0028) | 0.5394 (0.0025) | 0.4773 (0.0033) | 0.4271 (0.0045) |
| 5.00 | 0.8509 (0.0012) | 0.7359 (0.0017) | 0.6446 (0.0028) | 0.5733 (0.0026) | 0.5111 (0.0031) | 0.4599 (0.0042) |
| 6.00 | 0.8611 (0.0013) | 0.7547 (0.0017) | 0.6708 (0.0032) | 0.6030 (0.0029) | 0.5417 (0.0035) | 0.4902 (0.0047) |
| 7.00 | 0.8688 (0.0013) | 0.7700 (0.0017) | 0.6936 (0.0034) | 0.6282 (0.0029) | 0.5683 (0.0040) | 0.5169 (0.0053) |
| 8.00 | 0.8751 (0.0013) | 0.7832 (0.0018) | 0.7136 (0.0033) | 0.6514 (0.0030) | 0.5920 (0.0039) | 0.5411 (0.0052) |
| 9.00 | 0.8802 (0.0017) | 0.7939 (0.0023) | 0.7305 (0.0036) | 0.6710 (0.0032) | 0.6126 (0.0045) | 0.5620 (0.0064) |
| 10.00 | 0.8842 (0.0014) | 0.8036 (0.0022) | 0.7468 (0.0031) | 0.6899 (0.0030) | 0.6327 (0.0040) | 0.5836 (0.0063) |
| 12.00 | 0.8910 (0.0018) | 0.8194 (0.0025) | 0.7743 (0.0039) | 0.7227 (0.0033) | 0.6676 (0.0047) | 0.6182 (0.0071) |

**Table S16.** Self-referenced normalized amplitudes for the emission signal $[A_{em}(n)/A_{em}(1)]/[\text{sinc(n/10)/sinc(1/10)}]$ for each harmonic at different concentrations. Mean and standard deviation (in parentheses) for the evaluation partition (estimated with 125 measurements at each concentration).

## 9. Self-referenced delays

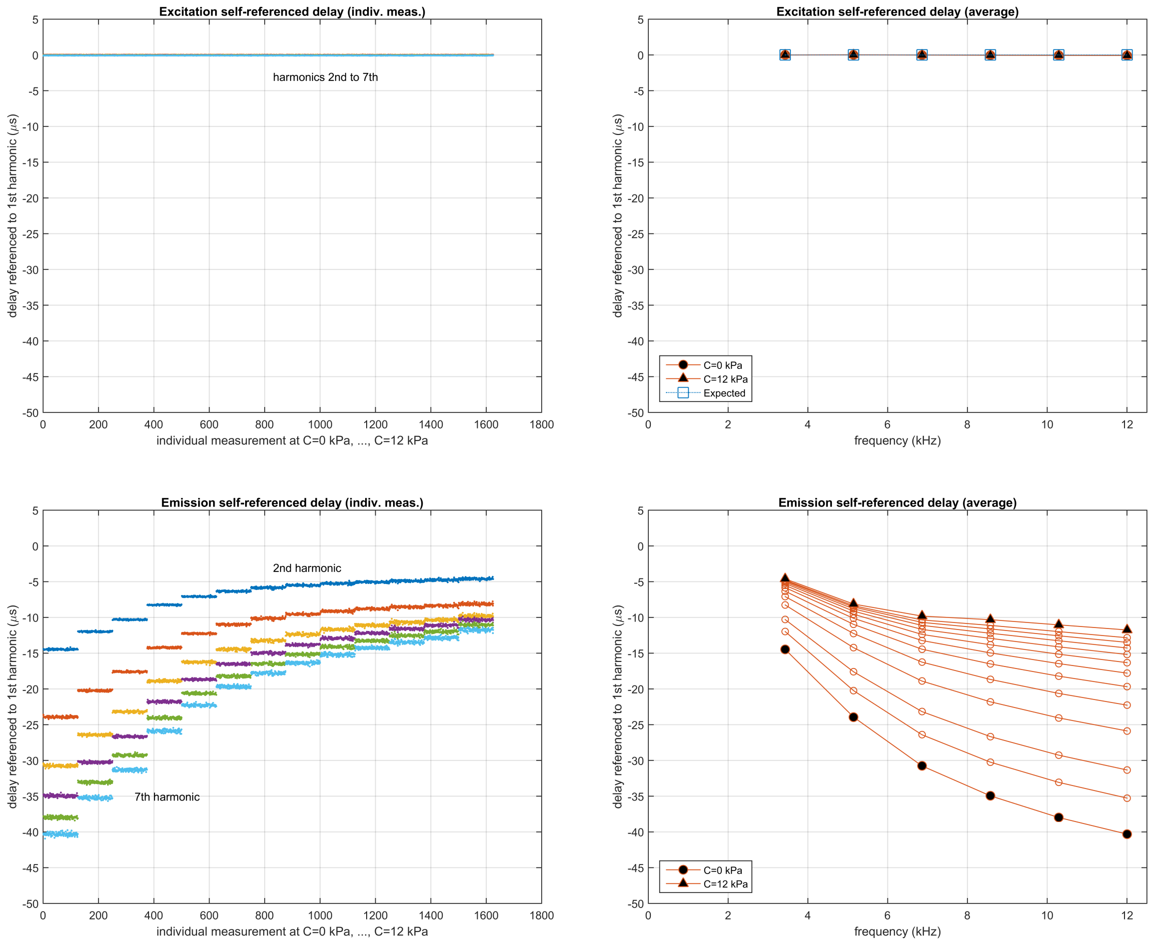


**Figure S19.** Self-referenced delay of the excitation signal (top) and the emission signal (bottom) for the calibration partition. First harmonic used as reference. The left panels show the individual measurements at increasing concentrations, from 0 to 12 kPa $pO_2$ (125 measurements for each concentration). The right panels show the average estimations.

| **Self-referenced delay for the excitation signal $\Delta t_{exc}(n)$ ($\mu$s) - Calibration partition** | | | | | | |
|---|---|---|---|---|---|---|
| $C$ (kPa) | $f$=3430 Hz ($n$=2) | $f$=5145 Hz ($n$=3) | $f$=6860 Hz ($n$=4) | $f$=8575 Hz ($n$=5) | $f$=10290 Hz ($n$=6) | $f$=12005 Hz ($n$=7) |
| 0.00 | -0.0008 (0.0119) | 0.0197 (0.0116) | -0.0112 (0.0113) | -0.0422 (0.0113) | -0.0603 (0.0115) | -0.0720 (0.0113) |
| 0.50 | -0.0007 (0.0111) | 0.0199 (0.0109) | -0.0105 (0.0102) | -0.0417 (0.0098) | -0.0594 (0.0109) | -0.0721 (0.0112) |
| 1.00 | -0.0007 (0.0106) | 0.0197 (0.0111) | -0.0089 (0.0112) | -0.0404 (0.0110) | -0.0578 (0.0103) | -0.0708 (0.0111) |
| 2.00 | -0.0033 (0.0113) | 0.0172 (0.0114) | -0.0110 (0.0110) | -0.0420 (0.0106) | -0.0591 (0.0106) | -0.0722 (0.0105) |
| 3.00 | -0.0033 (0.0115) | 0.0168 (0.0107) | -0.0111 (0.0109) | -0.0421 (0.0107) | -0.0597 (0.0108) | -0.0722 (0.0108) |
| 4.00 | -0.0066 (0.0102) | 0.0143 (0.0112) | -0.0130 (0.0100) | -0.0446 (0.0096) | -0.0623 (0.0097) | -0.0754 (0.0104) |
| 5.00 | -0.0043 (0.0113) | 0.0152 (0.0114) | -0.0123 (0.0116) | -0.0435 (0.0121) | -0.0602 (0.0115) | -0.0738 (0.0121) |
| 6.00 | -0.0050 (0.0118) | 0.0160 (0.0114) | -0.0110 (0.0114) | -0.0424 (0.0111) | -0.0593 (0.0113) | -0.0725 (0.0114) |
| 7.00 | -0.0066 (0.0110) | 0.0138 (0.0110) | -0.0130 (0.0106) | -0.0435 (0.0099) | -0.0605 (0.0110) | -0.0739 (0.0107) |
| 8.00 | -0.0089 (0.0126) | 0.0122 (0.0124) | -0.0151 (0.0116) | -0.0464 (0.0118) | -0.0636 (0.0118) | -0.0765 (0.0122) |
| 9.00 | -0.0097 (0.0132) | 0.0114 (0.0117) | -0.0155 (0.0119) | -0.0466 (0.0116) | -0.0638 (0.0117) | -0.0770 (0.0124) |
| 10.00 | -0.0066 (0.0119) | 0.0144 (0.0114) | -0.0133 (0.0113) | -0.0439 (0.0115) | -0.0608 (0.0114) | -0.0730 (0.0113) |
| 12.00 | -0.0058 (0.0126) | 0.0147 (0.0122) | -0.0126 (0.0112) | -0.0430 (0.0114) | -0.0606 (0.0115) | -0.0730 (0.0114) |
| Expected | 0 | 0 | 0 | 0 | 0 | 0 |

**Table S17.** Self-referenced delay for the excitation signal $\Delta t_{exc}(n)$ ($\mu$s) for each harmonic at different concentrations. Mean and standard deviation (in parentheses) for the calibration partition (estimated with 125 measurements at each concentration).

| **Self-referenced delay for the excitation signal $\Delta t_{exc}(n)$ ($\mu$s) - Evaluation partition** | | | | | | |
|---|---|---|---|---|---|---|
| $C$ (kPa) | $f$=3430 Hz ($n$=2) | $f$=5145 Hz ($n$=3) | $f$=6860 Hz ($n$=4) | $f$=8575 Hz ($n$=5) | $f$=10290 Hz ($n$=6) | $f$=12005 Hz ($n$=7) |
| 0.00 | -0.0016 (0.0113) | 0.0187 (0.0105) | -0.0116 (0.0099) | -0.0431 (0.0102) | -0.0604 (0.0102) | -0.0728 (0.0107) |
| 0.50 | 0.0003 (0.0116) | 0.0203 (0.0115) | -0.0098 (0.0116) | -0.0415 (0.0114) | -0.0592 (0.0109) | -0.0714 (0.0119) |
| 1.00 | -0.0019 (0.0117) | 0.0189 (0.0112) | -0.0101 (0.0113) | -0.0411 (0.0106) | -0.0590 (0.0110) | -0.0717 (0.0111) |
| 2.00 | -0.0037 (0.0124) | 0.0173 (0.0116) | -0.0120 (0.0115) | -0.0428 (0.0116) | -0.0600 (0.0112) | -0.0725 (0.0118) |
| 3.00 | -0.0012 (0.0120) | 0.0191 (0.0113) | -0.0094 (0.0113) | -0.0398 (0.0111) | -0.0572 (0.0114) | -0.0700 (0.0116) |
| 4.00 | -0.0049 (0.0109) | 0.0158 (0.0109) | -0.0110 (0.0101) | -0.0430 (0.0103) | -0.0602 (0.0097) | -0.0734 (0.0105) |
| 5.00 | -0.0065 (0.0115) | 0.0139 (0.0115) | -0.0138 (0.0122) | -0.0441 (0.0117) | -0.0617 (0.0117) | -0.0759 (0.0120) |
| 6.00 | -0.0068 (0.0116) | 0.0142 (0.0112) | -0.0130 (0.0108) | -0.0439 (0.0108) | -0.0617 (0.0107) | -0.0747 (0.0109) |
| 7.00 | -0.0078 (0.0122) | 0.0128 (0.0120) | -0.0146 (0.0120) | -0.0458 (0.0117) | -0.0625 (0.0114) | -0.0765 (0.0120) |
| 8.00 | -0.0077 (0.0112) | 0.0135 (0.0107) | -0.0147 (0.0103) | -0.0455 (0.0105) | -0.0630 (0.0103) | -0.0766 (0.0104) |
| 9.00 | -0.0060 (0.0105) | 0.0145 (0.0106) | -0.0122 (0.0098) | -0.0430 (0.0102) | -0.0604 (0.0101) | -0.0741 (0.0104) |
| 10.00 | -0.0093 (0.0109) | 0.0106 (0.0113) | -0.0159 (0.0110) | -0.0465 (0.0105) | -0.0634 (0.0106) | -0.0762 (0.0114) |
| 12.00 | -0.0088 (0.0112) | 0.0120 (0.0108) | -0.0155 (0.0098) | -0.0456 (0.0105) | -0.0629 (0.0106) | -0.0764 (0.0104) |
| Expected | 0 | 0 | 0 | 0 | 0 | 0 |

**Table S18.** Self-referenced delay for the excitation signal $\Delta t_{exc}(n)$ ($\mu$s) for each harmonic at different concentrations. Mean and standard deviation (in parentheses) for the evaluation partition (estimated with 125 measurements at each concentration).

| **Self-referenced delay for the emission signal $\Delta t_{em}(n)$ ($\mu$s) - Calibration partition** | | | | | | |
|---|---|---|---|---|---|---|
| $C$ (kPa) | $f$=3430 Hz ($n$=2) | $f$=5145 Hz ($n$=3) | $f$=6860 Hz ($n$=4) | $f$=8575 Hz ($n$=5) | $f$=10290 Hz ($n$=6) | $f$=12005 Hz ($n$=7) |
| 0.00 | -14.467 (0.084) | -23.924 (0.115) | -30.758 (0.143) | -34.950 (0.148) | -37.980 (0.166) | -40.312 (0.210) |
| 0.50 | -11.981 (0.069) | -20.235 (0.092) | -26.410 (0.117) | -30.251 (0.121) | -33.056 (0.137) | -35.246 (0.172) |
| 1.00 | -10.302 (0.065) | -17.598 (0.082) | -23.178 (0.113) | -26.665 (0.108) | -29.280 (0.130) | -31.335 (0.164) |
| 2.00 | -8.248 (0.064) | -14.224 (0.084) | -18.894 (0.120) | -21.790 (0.119) | -24.051 (0.137) | -25.885 (0.176) |
| 3.00 | -7.079 (0.065) | -12.255 (0.075) | -16.252 (0.119) | -18.676 (0.096) | -20.632 (0.118) | -22.274 (0.147) |
| 4.00 | -6.338 (0.080) | -10.982 (0.102) | -14.462 (0.136) | -16.506 (0.111) | -18.219 (0.134) | -19.680 (0.165) |
| 5.00 | -5.835 (0.102) | -10.128 (0.119) | -13.241 (0.143) | -14.971 (0.127) | -16.467 (0.132) | -17.787 (0.157) |
| 6.00 | -5.503 (0.093) | -9.547 (0.103) | -12.372 (0.144) | -13.824 (0.111) | -15.156 (0.133) | -16.352 (0.160) |
| 7.00 | -5.251 (0.105) | -9.127 (0.115) | -11.677 (0.153) | -12.926 (0.137) | -14.107 (0.152) | -15.197 (0.181) |
| 8.00 | -5.053 (0.095) | -8.793 (0.105) | -11.114 (0.137) | -12.202 (0.116) | -13.264 (0.130) | -14.267 (0.143) |
| 9.00 | -4.899 (0.110) | -8.547 (0.123) | -10.692 (0.161) | -11.598 (0.140) | -12.559 (0.153) | -13.477 (0.172) |
| 10.00 | -4.764 (0.118) | -8.362 (0.121) | -10.343 (0.138) | -11.111 (0.134) | -11.981 (0.155) | -12.848 (0.166) |
| 12.00 | -4.600 (0.107) | -8.121 (0.136) | -9.800 (0.161) | -10.323 (0.150) | -11.035 (0.157) | -11.757 (0.184) |

**Table S19.** Self-referenced delay for the emission signal $\Delta t_{em}(n)$ ($\mu$s) for each harmonic at different concentrations. Mean and standard deviation (in parentheses) for the calibration partition (estimated with 125 measurements at each concentration).

| **Self-referenced delay for the emission signal $\Delta t_{em}(n)$ ($\mu$s) - Evaluation partition** | | | | | | |
|---|---|---|---|---|---|---|
| $C$ (kPa) | $f$=3430 Hz ($n$=2) | $f$=5145 Hz ($n$=3) | $f$=6860 Hz ($n$=4) | $f$=8575 Hz ($n$=5) | $f$=10290 Hz ($n$=6) | $f$=12005 Hz ($n$=7) |
| 0.00 | -14.457 (0.068) | -23.908 (0.095) | -30.754 (0.120) | -34.907 (0.121) | -37.924 (0.158) | -40.254 (0.172) |
| 0.50 | -11.981 (0.070) | -20.240 (0.095) | -26.403 (0.131) | -30.244 (0.136) | -33.057 (0.155) | -35.259 (0.197) |
| 1.00 | -10.296 (0.061) | -17.585 (0.084) | -23.170 (0.124) | -26.671 (0.135) | -29.289 (0.162) | -31.354 (0.172) |
| 2.00 | -8.245 (0.063) | -14.228 (0.082) | -18.898 (0.116) | -21.794 (0.112) | -24.060 (0.122) | -25.895 (0.156) |
| 3.00 | -7.089 (0.075) | -12.265 (0.091) | -16.266 (0.134) | -18.678 (0.122) | -20.638 (0.143) | -22.272 (0.174) |
| 4.00 | -6.334 (0.080) | -10.971 (0.094) | -14.469 (0.134) | -16.504 (0.125) | -18.213 (0.137) | -19.673 (0.172) |
| 5.00 | -5.866 (0.099) | -10.154 (0.105) | -13.256 (0.137) | -14.976 (0.120) | -16.477 (0.138) | -17.802 (0.181) |
| 6.00 | -5.499 (0.109) | -9.528 (0.126) | -12.328 (0.154) | -13.808 (0.143) | -15.138 (0.148) | -16.330 (0.185) |
| 7.00 | -5.247 (0.100) | -9.118 (0.106) | -11.668 (0.142) | -12.936 (0.117) | -14.128 (0.131) | -15.212 (0.157) |
| 8.00 | -5.049 (0.112) | -8.791 (0.128) | -11.108 (0.161) | -12.193 (0.147) | -13.254 (0.149) | -14.251 (0.178) |
| 9.00 | -4.877 (0.107) | -8.536 (0.113) | -10.667 (0.158) | -11.595 (0.133) | -12.558 (0.156) | -13.474 (0.181) |
| 10.00 | -4.795 (0.103) | -8.390 (0.123) | -10.364 (0.135) | -11.137 (0.117) | -12.008 (0.133) | -12.863 (0.170) |
| 12.00 | -4.614 (0.114) | -8.136 (0.129) | -9.828 (0.145) | -10.329 (0.143) | -11.038 (0.147) | -11.797 (0.171) |

**Table S20.** Self-referenced delay for the emission signal $\Delta t_{em}(n)$ ($\mu$s) for each harmonic at different concentrations. Mean and standard deviation (in parentheses) for the evaluation partition (estimated with 125 measurements at each concentration).

## 10. Self-referenced apparent lifetimes

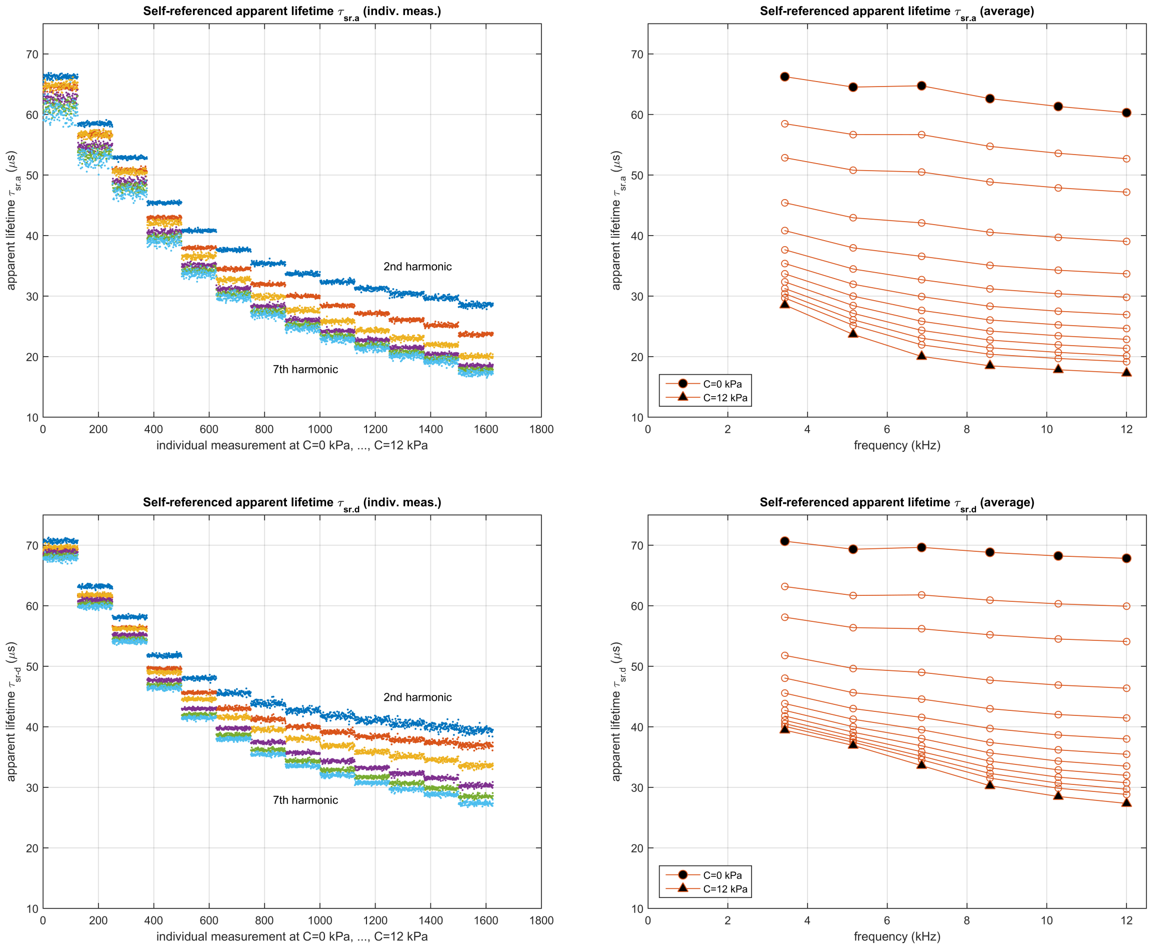


**Figure S20.** Apparent lifetime estimations based on the self-referenced normalized amplitudes (top) and the self-referenced delays (bottom) for the calibration partition. First harmonic used as reference. The left panels show the individual measurements at increasing concentrations, from 0 to 12 kPa $pO_2$ (125 measurements for each concentration). The right panels show the average estimations.

| **Apparent lifetimes estimated from self-ref. normalized amplitudes $\tau_{sr.a}$ ($\mu$s) - Calibration partition** | | | | | | |
|---|---|---|---|---|---|---|
| $C$ (kPa) | $f$=3430 Hz ($n$=2) | $f$=5145 Hz ($n$=3) | $f$=6860 Hz ($n$=4) | $f$=8575 Hz ($n$=5) | $f$=10290 Hz ($n$=6) | $f$=12005 Hz ($n$=7) |
| 0.00 | 66.250 (0.285) | 64.531 (0.375) | 64.746 (0.523) | 62.630 (0.572) | 61.335 (0.804) | 60.339 (1.267) |
| 0.50 | 58.480 (0.220) | 56.688 (0.273) | 56.677 (0.421) | 54.733 (0.450) | 53.603 (0.685) | 52.717 (0.966) |
| 1.00 | 52.858 (0.187) | 50.798 (0.243) | 50.499 (0.355) | 48.851 (0.400) | 47.883 (0.511) | 47.183 (0.726) |
| 2.00 | 45.414 (0.165) | 42.935 (0.210) | 42.095 (0.339) | 40.541 (0.297) | 39.696 (0.368) | 39.026 (0.528) |
| 3.00 | 40.810 (0.161) | 37.965 (0.158) | 36.563 (0.298) | 35.084 (0.263) | 34.273 (0.323) | 33.682 (0.464) |
| 4.00 | 37.643 (0.176) | 34.471 (0.179) | 32.705 (0.274) | 31.195 (0.223) | 30.383 (0.280) | 29.808 (0.441) |
| 5.00 | 35.387 (0.209) | 31.953 (0.175) | 29.911 (0.261) | 28.313 (0.216) | 27.502 (0.279) | 26.927 (0.409) |
| 6.00 | 33.669 (0.209) | 29.980 (0.176) | 27.630 (0.261) | 26.051 (0.214) | 25.250 (0.261) | 24.653 (0.358) |
| 7.00 | 32.346 (0.207) | 28.424 (0.173) | 25.835 (0.244) | 24.219 (0.187) | 23.447 (0.234) | 22.856 (0.323) |
| 8.00 | 31.240 (0.215) | 27.146 (0.173) | 24.331 (0.225) | 22.732 (0.191) | 21.936 (0.251) | 21.356 (0.350) |
| 9.00 | 30.394 (0.263) | 26.083 (0.189) | 23.038 (0.256) | 21.458 (0.194) | 20.700 (0.219) | 20.110 (0.306) |
| 10.00 | 29.682 (0.250) | 25.179 (0.214) | 21.948 (0.203) | 20.389 (0.186) | 19.689 (0.225) | 19.164 (0.373) |
| 12.00 | 28.533 (0.284) | 23.659 (0.197) | 20.032 (0.230) | 18.479 (0.187) | 17.831 (0.217) | 17.266 (0.291) |

**Table S21.** Apparent lifetimes estimated from the self-referenced normalized amplitudes $\tau_{sr.a}$ ($\mu$s) for each harmonic at different concentrations. Mean and standard deviation (in parentheses) for the calibration partition (estimated with 125 measurements at each concentration).

| **Apparent lifetimes estimated from self-ref. normalized amplitudes $\tau_{sr.a}$ ($\mu$s) - Evaluation partition** | | | | | | |
|---|---|---|---|---|---|---|
| $C$ (kPa) | $f$=3430 Hz ($n$=2) | $f$=5145 Hz ($n$=3) | $f$=6860 Hz ($n$=4) | $f$=8575 Hz ($n$=5) | $f$=10290 Hz ($n$=6) | $f$=12005 Hz ($n$=7) |
| 0.00 | 66.211 (0.256) | 64.476 (0.325) | 64.695 (0.494) | 62.446 (0.591) | 61.285 (0.748) | 60.303 (1.218) |
| 0.50 | 58.504 (0.235) | 56.704 (0.299) | 56.670 (0.439) | 54.837 (0.494) | 53.686 (0.634) | 52.849 (0.970) |
| 1.00 | 52.836 (0.207) | 50.785 (0.249) | 50.502 (0.388) | 48.876 (0.386) | 47.867 (0.448) | 47.112 (0.745) |
| 2.00 | 45.402 (0.176) | 42.950 (0.188) | 42.073 (0.326) | 40.561 (0.282) | 39.688 (0.353) | 39.063 (0.552) |
| 3.00 | 40.793 (0.184) | 37.964 (0.189) | 36.634 (0.307) | 35.085 (0.290) | 34.276 (0.364) | 33.714 (0.511) |
| 4.00 | 37.638 (0.192) | 34.482 (0.177) | 32.719 (0.267) | 31.196 (0.224) | 30.387 (0.299) | 29.786 (0.420) |
| 5.00 | 35.401 (0.207) | 31.929 (0.179) | 29.850 (0.242) | 28.306 (0.207) | 27.517 (0.250) | 26.930 (0.339) |
| 6.00 | 33.645 (0.221) | 29.973 (0.176) | 27.645 (0.264) | 26.027 (0.209) | 25.226 (0.251) | 24.648 (0.330) |
| 7.00 | 32.341 (0.221) | 28.438 (0.165) | 25.832 (0.264) | 24.248 (0.198) | 23.425 (0.257) | 22.846 (0.336) |
| 8.00 | 31.263 (0.229) | 27.143 (0.174) | 24.321 (0.247) | 22.717 (0.190) | 21.943 (0.233) | 21.363 (0.303) |
| 9.00 | 30.399 (0.297) | 26.108 (0.216) | 23.087 (0.256) | 21.487 (0.196) | 20.737 (0.254) | 20.178 (0.351) |
| 10.00 | 29.720 (0.236) | 25.181 (0.206) | 21.925 (0.217) | 20.351 (0.176) | 19.626 (0.215) | 19.028 (0.323) |
| 12.00 | 28.563 (0.300) | 23.693 (0.230) | 20.034 (0.263) | 18.470 (0.183) | 17.816 (0.237) | 17.328 (0.336) |

**Table S22.** Apparent lifetimes estimated from the self-referenced normalized amplitudes $\tau_{sr.a}$ ($\mu$s) for each harmonic at different concentrations. Mean and standard deviation (in parentheses) for the evaluation partition (estimated with 125 measurements at each concentration).

| **Apparent lifetimes estimated from self-ref. delays $\tau_{sr.d}$ ($\mu$s) - Calibration partition** | | | | | | |
|---|---|---|---|---|---|---|
| $C$ (kPa) | $f$=3430 Hz ($n$=2) | $f$=5145 Hz ($n$=3) | $f$=6860 Hz ($n$=4) | $f$=8575 Hz ($n$=5) | $f$=10290 Hz ($n$=6) | $f$=12005 Hz ($n$=7) |
| 0.00 | 70.660 (0.252) | 69.305 (0.243) | 69.636 (0.265) | 68.819 (0.257) | 68.220 (0.276) | 67.836 (0.342) |
| 0.50 | 63.192 (0.206) | 61.698 (0.187) | 61.801 (0.206) | 60.934 (0.198) | 60.291 (0.213) | 59.887 (0.260) |
| 1.00 | 58.126 (0.196) | 56.383 (0.165) | 56.210 (0.193) | 55.203 (0.169) | 54.530 (0.194) | 54.090 (0.238) |
| 2.00 | 51.783 (0.201) | 49.628 (0.168) | 48.992 (0.201) | 47.695 (0.180) | 46.891 (0.196) | 46.391 (0.242) |
| 3.00 | 48.035 (0.211) | 45.647 (0.154) | 44.585 (0.198) | 43.001 (0.145) | 42.042 (0.165) | 41.466 (0.198) |
| 4.00 | 45.575 (0.269) | 43.030 (0.212) | 41.591 (0.228) | 39.746 (0.167) | 38.656 (0.188) | 37.983 (0.220) |
| 5.00 | 43.860 (0.351) | 41.247 (0.251) | 39.531 (0.242) | 37.436 (0.192) | 36.202 (0.184) | 35.456 (0.210) |
| 6.00 | 42.702 (0.328) | 40.017 (0.220) | 38.053 (0.246) | 35.698 (0.168) | 34.362 (0.187) | 33.543 (0.213) |
| 7.00 | 41.805 (0.378) | 39.117 (0.247) | 36.860 (0.264) | 34.330 (0.210) | 32.883 (0.215) | 32.000 (0.242) |
| 8.00 | 41.092 (0.345) | 38.393 (0.228) | 35.886 (0.238) | 33.218 (0.179) | 31.687 (0.184) | 30.753 (0.192) |
| 9.00 | 40.530 (0.402) | 37.857 (0.269) | 35.148 (0.282) | 32.283 (0.218) | 30.683 (0.218) | 29.689 (0.232) |
| 10.00 | 40.033 (0.437) | 37.451 (0.267) | 34.535 (0.243) | 31.525 (0.210) | 29.853 (0.224) | 28.838 (0.226) |
| 12.00 | 39.419 (0.402) | 36.920 (0.303) | 33.574 (0.287) | 30.282 (0.238) | 28.483 (0.229) | 27.350 (0.252) |

**Table S23.** Apparent lifetimes estimated from the self-referenced delays $\tau_{sr.d}$ ($\mu$s) for each harmonic at different concentrations. Mean and standard deviation (in parentheses) for the calibration partition (estimated with 125 measurements at each concentration).

| **Apparent lifetimes estimated from self-ref. delays $\tau_{sr.d}$ ($\mu$s) - Evaluation partition** | | | | | | |
|---|---|---|---|---|---|---|
| $C$ (kPa) | $f$=3430 Hz ($n$=2) | $f$=5145 Hz ($n$=3) | $f$=6860 Hz ($n$=4) | $f$=8575 Hz ($n$=5) | $f$=10290 Hz ($n$=6) | $f$=12005 Hz ($n$=7) |
| 0.00 | 70.630 (0.206) | 69.272 (0.199) | 69.628 (0.222) | 68.744 (0.210) | 68.125 (0.263) | 67.743 (0.280) |
| 0.50 | 63.192 (0.210) | 61.708 (0.192) | 61.789 (0.229) | 60.922 (0.222) | 60.293 (0.241) | 59.906 (0.298) |
| 1.00 | 58.108 (0.185) | 56.358 (0.169) | 56.196 (0.211) | 55.212 (0.211) | 54.543 (0.243) | 54.117 (0.250) |
| 2.00 | 51.773 (0.199) | 49.635 (0.165) | 48.998 (0.193) | 47.701 (0.169) | 46.903 (0.175) | 46.405 (0.215) |
| 3.00 | 48.066 (0.244) | 45.668 (0.186) | 44.609 (0.224) | 43.005 (0.184) | 42.051 (0.201) | 41.464 (0.235) |
| 4.00 | 45.561 (0.271) | 43.007 (0.196) | 41.602 (0.225) | 39.742 (0.188) | 38.647 (0.192) | 37.974 (0.231) |
| 5.00 | 43.965 (0.342) | 41.302 (0.220) | 39.557 (0.233) | 37.443 (0.182) | 36.217 (0.194) | 35.476 (0.241) |
| 6.00 | 42.684 (0.386) | 39.977 (0.268) | 37.978 (0.264) | 35.675 (0.217) | 34.336 (0.209) | 33.513 (0.246) |
| 7.00 | 41.790 (0.358) | 39.097 (0.228) | 36.844 (0.245) | 34.346 (0.180) | 32.913 (0.186) | 32.019 (0.210) |
| 8.00 | 41.077 (0.405) | 38.390 (0.277) | 35.874 (0.279) | 33.205 (0.227) | 31.674 (0.212) | 30.731 (0.239) |
| 9.00 | 40.450 (0.394) | 37.833 (0.247) | 35.106 (0.278) | 32.279 (0.207) | 30.681 (0.223) | 29.685 (0.244) |
| 10.00 | 40.146 (0.381) | 37.514 (0.270) | 34.573 (0.238) | 31.564 (0.183) | 29.892 (0.191) | 28.858 (0.230) |
| 12.00 | 39.471 (0.427) | 36.952 (0.286) | 33.624 (0.258) | 30.292 (0.226) | 28.488 (0.214) | 27.406 (0.234) |

**Table S24.** Apparent lifetimes estimated from the self-referenced delays $\tau_{sr.d}$ ($\mu$s) for each harmonic at different concentrations. Mean and standard deviation (in parentheses) for the evaluation partition (estimated with 125 measurements at each concentration).

## 11. Calibration parameters for each oxygen determination

### *11.1. Parameters of the Demas models providing the curves* $C(\tau)$

The Demas model providing the calibration curves to obtain the concentration $C$ as a function of the apparent lifetime $\tau$ or vice-versa is given by the function:

$$\tau = \tau_0 \left( \frac{x}{1 + k_1 C} + \frac{1 - x}{1 + k_2 C} \right) \tag{5}$$

The following tables provide the Demas model parameters for the different lifetime definitions and harmonics included in the experiments:

| **Oxygen determination using the modulation factor based lifetime $\tau_m(n)$ (conventional method)** **Parameters of the Demas model:** | | | | | | | |
|---|---|---|---|---|---|---|---|
| harmonic | 1 | 2 | 3 | 4 | 5 | 6 | 7 |
| $\tau_0$ ($\mu$s) | 67.331 | 64.570 | 63.369 | 58.548 | 60.164 | 63.707 | 69.718 |
| $k_1$ (kPa$^{-1}$) | 0.3109 | 0.3333 | 0.3460 | 0.3167 | 0.3270 | 0.3612 | 0.4242 |
| $k_2$ (kPa$^{-1}$) | 0.0106 | 0.0000 | 0.0000 | 0.1222 | 0.0722 | 0.0617 | 0.0007 |
| $x$ | 0.9532 | 0.9652 | 0.9692 | 0.8858 | 0.9489 | 0.9632 | 0.9785 |
| $R^2$ | 0.9999912 | 0.9999522 | 0.9997368 | 0.9990425 | 0.9990570 | 0.9990149 | 0.9995249 |
| $K$ (kPa$^{-1}$) | 0.2968 | 0.3217 | 0.3353 | 0.2944 | 0.3140 | 0.3502 | 0.4151 |
| $m_0(n)$ | 1.8226 | 1.2841 | 0.9592 | 0.7499 | 0.6241 | 0.5321 | 0.4622 |
| $T_0(n)$ ($\mu$s) | 67.3100 | 63.9701 | 62.0802 | 57.0858 | 57.1504 | 58.7554 | 61.1508 |

The estimation of the apparent lifetime from the modulation factor requires the parameters $T_0(n)$ and $m_0(n)$:

$$\tau_m(n) = T_0(n) \frac{m(n)/m_0(n)}{\sqrt{1 + (n\,\omega_0 T_0(n))^2 (1 - [m(n)/m_0(n)]^2)}} \tag{6}$$

| **Oxygen determination using the phase-shift based lifetime $\tau_\phi(n)$ (conventional method)** **Parameters of the Demas model:** | | | | | | | |
|---|---|---|---|---|---|---|---|
| harmonic | 1 | 2 | 3 | 4 | 5 | 6 | 7 |
| $\tau_0$ ($\mu$s) | 67.322 | 64.320 | 62.544 | 57.507 | 57.588 | 59.552 | 62.454 |
| $k_1$ (kPa$^{-1}$) | 0.3728 | 0.3738 | 0.3640 | 0.3215 | 0.3266 | 0.3421 | 0.3618 |
| $k_2$ (kPa$^{-1}$) | 0.0165 | 0.0206 | 0.0323 | 0.0000 | 0.0000 | 0.0000 | 0.0000 |
| $x$ | 0.7445 | 0.8051 | 0.8635 | 0.9322 | 0.9241 | 0.9256 | 0.9316 |
| $R^2$ | 0.9999996 | 0.9999983 | 0.9999920 | 0.9999816 | 0.9999337 | 0.9999277 | 0.9998202 |
| $K$ (kPa$^{-1}$) | 0.2817 | 0.3050 | 0.3187 | 0.2997 | 0.3018 | 0.3166 | 0.3371 |

| **Oxygen determination using the norm. amplitude based lifetime $\tau_{sr.a}(n)$ (self-ref. method)** **Parameters of the Demas model:** | | | | | | |
|---|---|---|---|---|---|---|
| harmonic | 2 | 3 | 4 | 5 | 6 | 7 |
| $\tau_0$ ($\mu$s) | 66.791 | 65.039 | 65.363 | 62.839 | 61.368 | 60.224 |
| $k_1$ (kPa$^{-1}$) | 0.4456 | 0.4146 | 0.4020 | 0.3610 | 0.3383 | 0.3245 |
| $k_2$ (kPa$^{-1}$) | 0.0000 | 0.0060 | 0.0232 | 0.0169 | 0.0071 | 0.0026 |
| $x$ | 0.6806 | 0.7430 | 0.7779 | 0.8331 | 0.8704 | 0.8910 |
| $R^2$ | 0.9999802 | 0.9999978 | 0.9999889 | 0.9999862 | 0.9999854 | 0.9999677 |
| $K$ (kPa$^{-1}$) | 0.3033 | 0.3096 | 0.3179 | 0.3036 | 0.2954 | 0.2894 |

| **Oxygen determination using the delay based lifetime $\tau_{sr.d}(n)$ (self-ref. method)** **Parameters of the Demas model:** | | | | | | |
|---|---|---|---|---|---|---|
| harmonic | 2 | 3 | 4 | 5 | 6 | 7 |
| $\tau_0$ ($\mu$s) | 71.982 | 71.703 | 70.615 | 69.171 | 68.501 | 68.084 |
| $k_1$ (kPa$^{-1}$) | 0.6123 | 0.6627 | 0.5089 | 0.4323 | 0.4141 | 0.4053 |
| $k_2$ (kPa$^{-1}$) | 0.0000 | 0.0000 | 0.0000 | 0.0000 | 0.0013 | 0.0030 |
| $x$ | 0.5157 | 0.5498 | 0.6114 | 0.6703 | 0.6958 | 0.7087 |
| $R^2$ | 0.9996314 | 0.9990188 | 0.9998973 | 0.9999896 | 0.9999959 | 0.9999951 |
| $K$ (kPa$^{-1}$) | 0.3158 | 0.3643 | 0.3111 | 0.2898 | 0.2885 | 0.2881 |

### *11.2. Uncertainties for each oxygen determination*

The following tables provide the RMS error (observed with the calibration partition) for each oxygen determination (for the different lifetime estimations and harmonics). These RMS errors are used as uncertainties of the individual oxygen determination in order to combine several individual determinations into a robust oxygen determination (the individual determinations are averaged using weights proportional to the squared inverse of the uncertainties).

| **Oxygen determination using the modulation factor based lifetime $\tau_m(n)$ (conventional method)** **RMS error in $pO_2$ determination (kPa) - Calibration partition** | | | | | | | |
|---|---|---|---|---|---|---|---|
| | harmonic | | | | | | |
| $pO_2$ (kPa) | 1 | 2 | 3 | 4 | 5 | 6 | 7 |
| 0.00 | 0.0085 | 0.0351 | 0.0783 | 0.1457 | 0.2636 | 0.4717 | 0.9153 |
| 0.50 | 0.0096 | 0.0183 | 0.0413 | 0.1041 | 0.1790 | 0.3477 | 0.5654 |
| 1.00 | 0.0118 | 0.0263 | 0.0576 | 0.1186 | 0.1769 | 0.3141 | 0.4931 |
| 2.00 | 0.0147 | 0.0212 | 0.0424 | 0.0992 | 0.1412 | 0.2442 | 0.4537 |
| 3.00 | 0.0177 | 0.0292 | 0.0467 | 0.1035 | 0.1322 | 0.2036 | 0.3850 |
| 4.00 | 0.0246 | 0.0368 | 0.0668 | 0.1430 | 0.1519 | 0.2082 | 0.3829 |
| 5.00 | 0.0247 | 0.0371 | 0.0764 | 0.1862 | 0.1847 | 0.2355 | 0.3659 |
| 6.00 | 0.0303 | 0.0385 | 0.0749 | 0.1727 | 0.1848 | 0.2322 | 0.3334 |
| 7.00 | 0.0333 | 0.0393 | 0.0651 | 0.1491 | 0.1533 | 0.1993 | 0.3052 |
| 8.00 | 0.0422 | 0.0493 | 0.0612 | 0.1000 | 0.1262 | 0.1864 | 0.3299 |
| 9.00 | 0.0476 | 0.0596 | 0.0683 | 0.1188 | 0.1306 | 0.1872 | 0.3197 |
| 10.00 | 0.0539 | 0.0637 | 0.0994 | 0.2051 | 0.1892 | 0.2145 | 0.3788 |
| 12.00 | 0.0718 | 0.0694 | 0.1346 | 0.4839 | 0.4618 | 0.4689 | 0.4277 |

| **Oxygen determination using the phase-shift based lifetime $\tau_{\phi}(n)$ (conventional method) RMS error in $pO_2$ determination (kPa) - Calibration partition** | | | | | | | |
|---|---|---|---|---|---|---|---|
| | harmonic | | | | | | |
| $pO_2$ (kPa) | 1 | 2 | 3 | 4 | 5 | 6 | 7 |
| 0.00 | 0.0078 | 0.0224 | 0.0326 | 0.0477 | 0.0710 | 0.1164 | 0.2120 |
| 0.50 | 0.0086 | 0.0136 | 0.0235 | 0.0454 | 0.0630 | 0.1002 | 0.1912 |
| 1.00 | 0.0112 | 0.0156 | 0.0272 | 0.0531 | 0.0788 | 0.1317 | 0.2167 |
| 2.00 | 0.0172 | 0.0228 | 0.0351 | 0.0679 | 0.0856 | 0.1351 | 0.2296 |
| 3.00 | 0.0255 | 0.0302 | 0.0410 | 0.0757 | 0.0817 | 0.1161 | 0.2114 |
| 4.00 | 0.0343 | 0.0332 | 0.0437 | 0.0751 | 0.0847 | 0.1370 | 0.2182 |
| 5.00 | 0.0543 | 0.0487 | 0.0541 | 0.0893 | 0.0952 | 0.1434 | 0.2326 |
| 6.00 | 0.0648 | 0.0631 | 0.0733 | 0.1217 | 0.1261 | 0.1767 | 0.2730 |
| 7.00 | 0.0885 | 0.0751 | 0.0758 | 0.1222 | 0.1361 | 0.2026 | 0.3109 |
| 8.00 | 0.0896 | 0.0811 | 0.0989 | 0.1595 | 0.1574 | 0.2297 | 0.3369 |
| 9.00 | 0.1198 | 0.0979 | 0.1107 | 0.1766 | 0.1708 | 0.2337 | 0.3257 |
| 10.00 | 0.1326 | 0.1044 | 0.1220 | 0.1552 | 0.1923 | 0.2576 | 0.3807 |
| 12.00 | 0.1783 | 0.1396 | 0.1612 | 0.2390 | 0.2167 | 0.3044 | 0.4780 |

| **Oxygen determination using the norm. amplitude based lifetime $\tau_{sr.a}(n)$ (self-referenced method) RMS error in $pO_2$ determination (kPa) - Calibration partition** | | | | | | |
|---|---|---|---|---|---|---|
| | harmonic | | | | | |
| $pO_2$ (kPa) | 2 | 3 | 4 | 5 | 6 | 7 |
| 0.00 | 0.0307 | 0.0319 | 0.0398 | 0.0321 | 0.0441 | 0.0717 |
| 0.50 | 0.0164 | 0.0197 | 0.0290 | 0.0328 | 0.0515 | 0.0750 |
| 1.00 | 0.0207 | 0.0239 | 0.0333 | 0.0388 | 0.0505 | 0.0731 |
| 2.00 | 0.0300 | 0.0345 | 0.0516 | 0.0453 | 0.0568 | 0.0828 |
| 3.00 | 0.0436 | 0.0385 | 0.0677 | 0.0585 | 0.0713 | 0.1034 |
| 4.00 | 0.0681 | 0.0619 | 0.0870 | 0.0688 | 0.0857 | 0.1328 |
| 5.00 | 0.1079 | 0.0787 | 0.1030 | 0.0848 | 0.1094 | 0.1600 |
| 6.00 | 0.1414 | 0.1007 | 0.1289 | 0.1046 | 0.1286 | 0.1766 |
| 7.00 | 0.1737 | 0.1227 | 0.1471 | 0.1120 | 0.1417 | 0.1962 |
| 8.00 | 0.2315 | 0.1479 | 0.1616 | 0.1376 | 0.1820 | 0.2539 |
| 9.00 | 0.3304 | 0.1922 | 0.2154 | 0.1657 | 0.1897 | 0.2666 |
| 10.00 | 0.3614 | 0.2510 | 0.1984 | 0.1896 | 0.2357 | 0.3874 |
| 12.00 | 0.5638 | 0.3054 | 0.2913 | 0.2514 | 0.2991 | 0.4076 |

| **Oxygen determination using the delay based lifetime $\tau_{sr.d}(n)$ (self-referenced method) RMS error in $pO_2$ determination (kPa) - Calibration partition** | | | | | | |
|---|---|---|---|---|---|---|
| | harmonic | | | | | |
| $pO_2$ (kPa) | 2 | 3 | 4 | 5 | 6 | 7 |
| 0.00 | 0.0616 | 0.0984 | 0.0474 | 0.0220 | 0.0202 | 0.0217 |
| 0.50 | 0.0169 | 0.0185 | 0.0153 | 0.0145 | 0.0157 | 0.0194 |
| 1.00 | 0.0350 | 0.0440 | 0.0253 | 0.0174 | 0.0196 | 0.0239 |
| 2.00 | 0.0656 | 0.0859 | 0.0461 | 0.0313 | 0.0333 | 0.0402 |
| 3.00 | 0.0799 | 0.0768 | 0.0589 | 0.0380 | 0.0422 | 0.0495 |
| 4.00 | 0.1438 | 0.1112 | 0.1009 | 0.0646 | 0.0669 | 0.0762 |
| 5.00 | 0.2839 | 0.2263 | 0.1493 | 0.0970 | 0.0876 | 0.0961 |
| 6.00 | 0.3447 | 0.2871 | 0.1880 | 0.1078 | 0.1120 | 0.1229 |
| 7.00 | 0.5005 | 0.3745 | 0.2566 | 0.1693 | 0.1611 | 0.1736 |
| 8.00 | 0.5529 | 0.4343 | 0.2955 | 0.1776 | 0.1684 | 0.1671 |
| 9.00 | 0.7680 | 0.5435 | 0.4045 | 0.2613 | 0.2415 | 0.2416 |
| 10.00 | 1.0533 | 0.5944 | 0.4101 | 0.2964 | 0.2884 | 0.2743 |
| 12.00 | 1.2329 | 1.0742 | 0.6667 | 0.4544 | 0.3966 | 0.4036 |

## 12. RMS error in oxygen determination (evaluation partition)

### *12.1. Not combined oxygen determination*

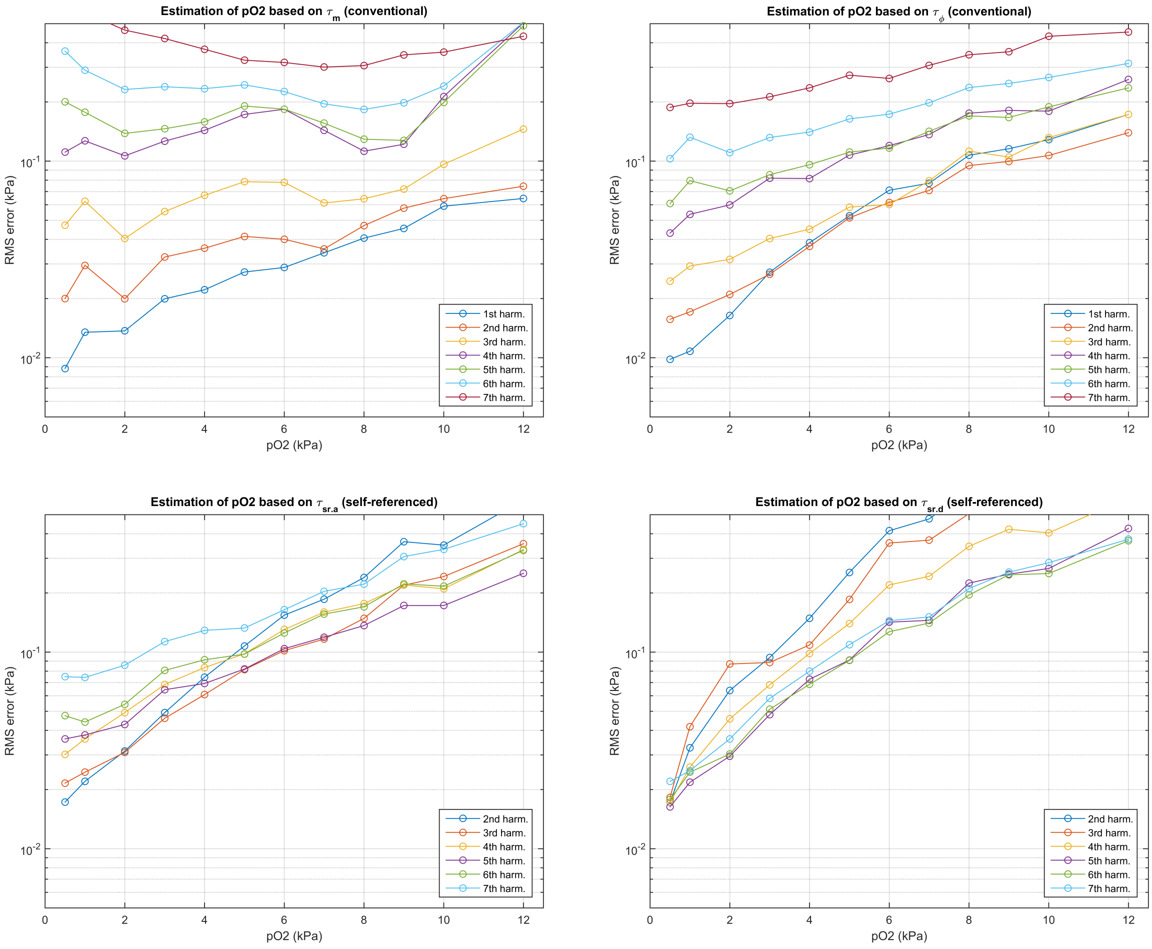


**Figure S21.** RMS error in oxygen determination (evaluation partition) as a function of the concentration. Conventional (top panels) and self-referenced (bottom panels) procedures for apparent lifetime estimation. Lifetime estimations based on modulation factor, phase-shift, self-referenced normalized amplitude and self-referenced delay.

| **Oxygen determination using the modulation factor based lifetime $\tau_m(n)$ (conventional method)**<br>**RMS error in $pO_2$ determination (kPa) - Evaluation partition** | | | | | | | |
|---|---|---|---|---|---|---|---|
| | harmonic | | | | | | |
| $pO_2$ (kPa) | 1 | 2 | 3 | 4 | 5 | 6 | 7 |
| 0.50 | 0.0088 | 0.0200 | 0.0472 | 0.1111 | 0.2005 | 0.3625 | 0.6001 |
| 1.00 | 0.0135 | 0.0295 | 0.0625 | 0.1268 | 0.1771 | 0.2900 | 0.5707 |
| 2.00 | 0.0137 | 0.0200 | 0.0404 | 0.1064 | 0.1383 | 0.2314 | 0.4636 |
| 3.00 | 0.0199 | 0.0325 | 0.0554 | 0.1264 | 0.1461 | 0.2389 | 0.4203 |
| 4.00 | 0.0222 | 0.0360 | 0.0670 | 0.1434 | 0.1584 | 0.2337 | 0.3706 |
| 5.00 | 0.0273 | 0.0414 | 0.0786 | 0.1728 | 0.1906 | 0.2442 | 0.3261 |
| 6.00 | 0.0288 | 0.0400 | 0.0779 | 0.1836 | 0.1838 | 0.2257 | 0.3175 |
| 7.00 | 0.0342 | 0.0358 | 0.0612 | 0.1435 | 0.1559 | 0.1955 | 0.3007 |
| 8.00 | 0.0406 | 0.0470 | 0.0643 | 0.1123 | 0.1293 | 0.1830 | 0.3062 |
| 9.00 | 0.0455 | 0.0576 | 0.0721 | 0.1218 | 0.1274 | 0.1982 | 0.3471 |
| 10.00 | 0.0590 | 0.0645 | 0.0964 | 0.2131 | 0.1989 | 0.2409 | 0.3583 |
| 12.00 | 0.0646 | 0.0745 | 0.1456 | 0.5052 | 0.4882 | 0.5079 | 0.4316 |
| average | 0.0315 | 0.0416 | 0.0724 | 0.1722 | 0.1912 | 0.2627 | 0.4011 |

**Table S25.** Accuracy in the oxygen determination using the modulation factor based lifetime (conventional method, individual determinations).

| **Oxygen determination using the phase-shift based lifetime $\tau_\phi(n)$ (conventional method)**<br>**RMS error in $pO_2$ determination (kPa) - Evaluation partition** | | | | | | | |
|---|---|---|---|---|---|---|---|
| | harmonic | | | | | | |
| $pO_2$ (kPa) | 1 | 2 | 3 | 4 | 5 | 6 | 7 |
| 0.50 | 0.0098 | 0.0157 | 0.0245 | 0.0431 | 0.0609 | 0.1029 | 0.1877 |
| 1.00 | 0.0108 | 0.0171 | 0.0293 | 0.0537 | 0.0796 | 0.1323 | 0.1969 |
| 2.00 | 0.0164 | 0.0210 | 0.0317 | 0.0599 | 0.0706 | 0.1105 | 0.1960 |
| 3.00 | 0.0272 | 0.0266 | 0.0404 | 0.0819 | 0.0853 | 0.1319 | 0.2123 |
| 4.00 | 0.0385 | 0.0368 | 0.0450 | 0.0815 | 0.0959 | 0.1405 | 0.2359 |
| 5.00 | 0.0527 | 0.0515 | 0.0584 | 0.1073 | 0.1111 | 0.1639 | 0.2736 |
| 6.00 | 0.0711 | 0.0616 | 0.0601 | 0.1199 | 0.1166 | 0.1733 | 0.2634 |
| 7.00 | 0.0772 | 0.0710 | 0.0793 | 0.1366 | 0.1418 | 0.1980 | 0.3070 |
| 8.00 | 0.1071 | 0.0950 | 0.1123 | 0.1753 | 0.1698 | 0.2366 | 0.3474 |
| 9.00 | 0.1156 | 0.0996 | 0.1049 | 0.1811 | 0.1667 | 0.2483 | 0.3598 |
| 10.00 | 0.1286 | 0.1068 | 0.1316 | 0.1795 | 0.1887 | 0.2662 | 0.4313 |
| 12.00 | 0.1728 | 0.1396 | 0.1729 | 0.2601 | 0.2357 | 0.3139 | 0.4533 |
| average | 0.0690 | 0.0618 | 0.0742 | 0.1233 | 0.1269 | 0.1849 | 0.2887 |

**Table S26.** Accuracy in the oxygen determination using the phase-shift based lifetime (conventional method, individual determinations).

| **Oxygen determination using the norm. amplitude based lifetime $\tau_{sr.a}(n)$ (self-referenced method)**<br>**RMS error in $pO_2$ determination (kPa) - Evaluation partition** | | | | | | |
|---|---|---|---|---|---|---|
| | harmonic | | | | | |
| $pO_2$ (kPa) | 2 | 3 | 4 | 5 | 6 | 7 |
| 0.50 | 0.0173 | 0.0216 | 0.0302 | 0.0362 | 0.0475 | 0.0750 |
| 1.00 | 0.0220 | 0.0245 | 0.0362 | 0.0379 | 0.0441 | 0.0743 |
| 2.00 | 0.0314 | 0.0309 | 0.0493 | 0.0429 | 0.0543 | 0.0858 |
| 3.00 | 0.0492 | 0.0461 | 0.0684 | 0.0644 | 0.0807 | 0.1131 |
| 4.00 | 0.0746 | 0.0608 | 0.0835 | 0.0692 | 0.0914 | 0.1290 |
| 5.00 | 0.1074 | 0.0814 | 0.0980 | 0.0820 | 0.0977 | 0.1326 |
| 6.00 | 0.1541 | 0.1018 | 0.1304 | 0.1041 | 0.1251 | 0.1642 |
| 7.00 | 0.1859 | 0.1165 | 0.1597 | 0.1190 | 0.1562 | 0.2039 |
| 8.00 | 0.2395 | 0.1484 | 0.1764 | 0.1365 | 0.1700 | 0.2217 |
| 9.00 | 0.3644 | 0.2190 | 0.2193 | 0.1725 | 0.2223 | 0.3063 |
| 10.00 | 0.3502 | 0.2423 | 0.2099 | 0.1726 | 0.2163 | 0.3331 |
| 12.00 | 0.5938 | 0.3561 | 0.3324 | 0.2517 | 0.3296 | 0.4499 |
| average | 0.1825 | 0.1208 | 0.1328 | 0.1074 | 0.1363 | 0.1907 |

**Table S27.** Accuracy in the oxygen determination using the self-referenced normalized amplitude based lifetime (self-referenced method, individual determinations).

| **Oxygen determination using the delay based lifetime $\tau_{sr.d}(n)$ (self-referenced method)**<br>**RMS error in $pO_2$ determination (kPa) - Evaluation partition** | | | | | | |
|---|---|---|---|---|---|---|
| | harmonic | | | | | |
| $pO_2$ (kPa) | 2 | 3 | 4 | 5 | 6 | 7 |
| 0.50 | 0.0172 | 0.0182 | 0.0172 | 0.0163 | 0.0178 | 0.0220 |
| 1.00 | 0.0326 | 0.0418 | 0.0260 | 0.0218 | 0.0245 | 0.0250 |
| 2.00 | 0.0639 | 0.0870 | 0.0458 | 0.0296 | 0.0303 | 0.0362 |
| 3.00 | 0.0939 | 0.0886 | 0.0682 | 0.0481 | 0.0512 | 0.0582 |
| 4.00 | 0.1486 | 0.1087 | 0.0985 | 0.0728 | 0.0686 | 0.0800 |
| 5.00 | 0.2544 | 0.1853 | 0.1398 | 0.0911 | 0.0908 | 0.1091 |
| 6.00 | 0.4144 | 0.3588 | 0.2196 | 0.1420 | 0.1270 | 0.1445 |
| 7.00 | 0.4760 | 0.3709 | 0.2430 | 0.1450 | 0.1405 | 0.1510 |
| 8.00 | 0.6618 | 0.5055 | 0.3453 | 0.2243 | 0.1954 | 0.2106 |
| 9.00 | 0.8438 | 0.5373 | 0.4215 | 0.2493 | 0.2472 | 0.2550 |
| 10.00 | 0.8642 | 0.6004 | 0.4042 | 0.2663 | 0.2510 | 0.2850 |
| 12.00 | 1.2617 | 1.0563 | 0.6097 | 0.4255 | 0.3687 | 0.3752 |
| average | 0.4277 | 0.3299 | 0.2199 | 0.1443 | 0.1344 | 0.1460 |

**Table S28.** Accuracy in the oxygen determination using the self-referenced delay based lifetime (self-referenced method, individual determinations).

## 12.2. Combined oxygen determination

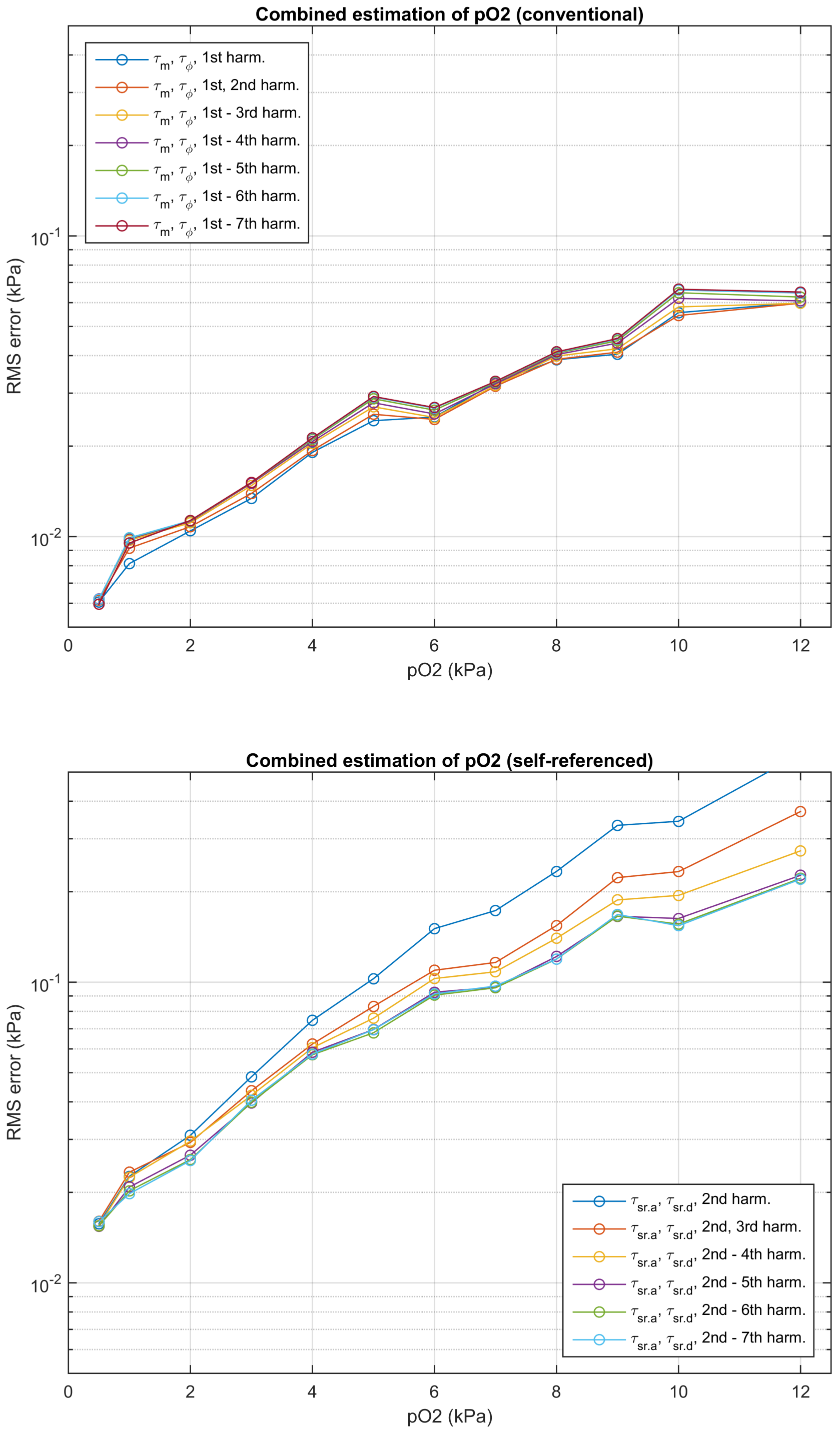


**Figure S22.** RMS error in oxygen determination (evaluation partition) as a function of the concentration. Top panel: conventional method combining $\tau_m(n)$ and $\tau_\phi(n)$ up to harmonic $n$. Bottom panel: self-referenced method combining $\tau_{sr.a}(n)$ and $\tau_{sr.d}(n)$ up to harmonic $n$.

| **Oxygen determination combining $\tau_m(n)$ and $\tau_\phi(n)$ (conventional method)**<br>**RMS error in $pO_2$ determination (kPa) - Evaluation partition** | | | | | | | |
|---|---|---|---|---|---|---|---|
| | harmonic | | | | | | |
| $pO_2$ (kPa) | 1 | 1,2 | 1,2,3 | 1,2,3,4 | 1,2,3,4,5 | 1,2,3,4,5,6 | 1,2,3,4,5,6,7 |
| 0.50 | 0.0061 | 0.0061 | 0.0062 | 0.0062 | 0.0062 | 0.0062 | 0.0060 |
| 1.00 | 0.0081 | 0.0092 | 0.0097 | 0.0098 | 0.0099 | 0.0099 | 0.0095 |
| 2.00 | 0.0104 | 0.0108 | 0.0111 | 0.0112 | 0.0113 | 0.0113 | 0.0113 |
| 3.00 | 0.0134 | 0.0139 | 0.0148 | 0.0150 | 0.0151 | 0.0151 | 0.0152 |
| 4.00 | 0.0191 | 0.0193 | 0.0204 | 0.0207 | 0.0210 | 0.0212 | 0.0213 |
| 5.00 | 0.0243 | 0.0255 | 0.0270 | 0.0279 | 0.0287 | 0.0291 | 0.0292 |
| 6.00 | 0.0250 | 0.0245 | 0.0249 | 0.0256 | 0.0263 | 0.0268 | 0.0269 |
| 7.00 | 0.0325 | 0.0316 | 0.0318 | 0.0322 | 0.0326 | 0.0328 | 0.0328 |
| 8.00 | 0.0388 | 0.0389 | 0.0398 | 0.0403 | 0.0408 | 0.0410 | 0.0412 |
| 9.00 | 0.0404 | 0.0411 | 0.0422 | 0.0440 | 0.0448 | 0.0454 | 0.0456 |
| 10.00 | 0.0556 | 0.0544 | 0.0581 | 0.0620 | 0.0647 | 0.0661 | 0.0665 |
| 12.00 | 0.0600 | 0.0598 | 0.0597 | 0.0608 | 0.0626 | 0.0646 | 0.0651 |
| average | 0.0278 | 0.0279 | 0.0288 | 0.0296 | 0.0303 | 0.0308 | 0.0309 |

**Table S29.** Accuracy in the oxygen determination combining the modulation factor and phase-shift based lifetimes from different harmonics (conventional method, combined determinations).

| **Oxygen determination combining $\tau_{sr.a}$ and $\tau_{sr.d}(n)$ (self-referenced method)**<br>**RMS error in $pO_2$ determination (kPa) - Evaluation partition** | | | | | | |
|---|---|---|---|---|---|---|
| | harmonic | | | | | |
| $pO_2$ (kPa) | 2 | 2,3 | 2,3,4 | 2,3,4,5 | 2,3,4,5,6 | 2,3,4,5,6,7 |
| 0.50 | 0.0157 | 0.0160 | 0.0155 | 0.0154 | 0.0155 | 0.0160 |
| 1.00 | 0.0226 | 0.0233 | 0.0224 | 0.0208 | 0.0202 | 0.0198 |
| 2.00 | 0.0310 | 0.0293 | 0.0296 | 0.0266 | 0.0257 | 0.0255 |
| 3.00 | 0.0485 | 0.0435 | 0.0420 | 0.0397 | 0.0399 | 0.0405 |
| 4.00 | 0.0747 | 0.0623 | 0.0605 | 0.0585 | 0.0574 | 0.0577 |
| 5.00 | 0.1027 | 0.0831 | 0.0759 | 0.0696 | 0.0679 | 0.0695 |
| 6.00 | 0.1507 | 0.1096 | 0.1029 | 0.0926 | 0.0905 | 0.0915 |
| 7.00 | 0.1731 | 0.1163 | 0.1083 | 0.0963 | 0.0958 | 0.0971 |
| 8.00 | 0.2336 | 0.1543 | 0.1400 | 0.1218 | 0.1193 | 0.1194 |
| 9.00 | 0.3325 | 0.2228 | 0.1878 | 0.1657 | 0.1659 | 0.1680 |
| 10.00 | 0.3431 | 0.2333 | 0.1945 | 0.1630 | 0.1561 | 0.1541 |
| 12.00 | 0.5737 | 0.3697 | 0.2735 | 0.2268 | 0.2217 | 0.2202 |
| average | 0.1752 | 0.1220 | 0.1044 | 0.0914 | 0.0897 | 0.0899 |

**Table S30.** Accuracy in the oxygen determination combining the self-referenced amplitude and delay based lifetimes from different harmonics (self-referenced method, combined determinations).

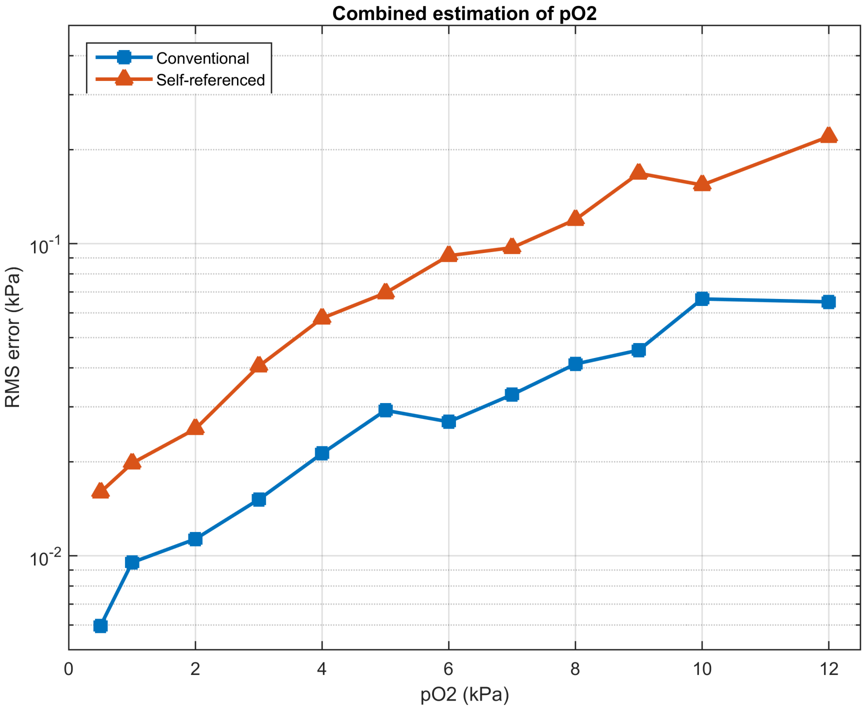


**Figure S23.** Comparison of the conventional and self-referenced methods for oxygen determination combining all the available lifetimes in each case.

**Oxygen determination combining lifetimes (comparison conventional vs. self-referenced methods)**
**RMS error in $pO_2$ determination (kPa) - Evaluation partition (in parenthesis the relative RMS error)**

| $pO_2$ (kPa) | conventional harm. 1-7 | | self-referenced harm. 2-7 | | self-ref. / conv. ratio |
|---|---|---|---|---|---|
| 0.50 | 0.0060 kPa | (1.20 %) | 0.0160 kPa | (3.20 %) | 2.6821 |
| 1.00 | 0.0095 kPa | (0.95 %) | 0.0198 kPa | (1.98 %) | 2.0802 |
| 2.00 | 0.0113 kPa | (0.56 %) | 0.0255 kPa | (1.27 %) | 2.2533 |
| 3.00 | 0.0152 kPa | (0.51 %) | 0.0405 kPa | (1.35 %) | 2.6721 |
| 4.00 | 0.0213 kPa | (0.53 %) | 0.0577 kPa | (1.44 %) | 2.7113 |
| 5.00 | 0.0292 kPa | (0.58 %) | 0.0695 kPa | (1.39 %) | 2.3772 |
| 6.00 | 0.0269 kPa | (0.45 %) | 0.0915 kPa | (1.52 %) | 3.4052 |
| 7.00 | 0.0328 kPa | (0.47 %) | 0.0971 kPa | (1.39 %) | 2.9574 |
| 8.00 | 0.0412 kPa | (0.51 %) | 0.1194 kPa | (1.49 %) | 2.9020 |
| 9.00 | 0.0456 kPa | (0.51 %) | 0.1680 kPa | (1.87 %) | 3.6866 |
| 10.00 | 0.0665 kPa | (0.66 %) | 0.1541 kPa | (1.54 %) | 2.3178 |
| 12.00 | 0.0651 kPa | (0.54 %) | 0.2202 kPa | (1.83 %) | 3.3843 |
| average | 0.0309 kPa | (0.62 %) | 0.0899 kPa | (1.69 %) | 2.79 |

**Table S31.** Accuracy in the oxygen determination combining the lifetimes from all the harmonics: comparison of the conventional and the self-referenced methods.
